# Artificial Intelligence-Based Wearable Sensing Technologies for the Management of Cancer, Diabetes, and COVID-19

**DOI:** 10.3390/bios15110756

**Published:** 2025-11-13

**Authors:** Amit Kumar, Shubham Goel, Abhishek Chaudhary, Sunil Dutt, Vivek K. Mishra, Raj Kumar

**Affiliations:** 1 Department of Computer Science and Engineering, Jaypee University of Information Technology, Waknaghat, Solan 173234, Himachal Pradesh, India; amit.kumar@juitsolan.in; 2 Department of Computer Science and Engineering, Gurugram University, Gurugram 122003, Haryana, India; shubhamgoel@gurugramuniversity.ac.in; 3 Department of Biotechnology and Bioinformatics, Jaypee University of Information Technology, Waknaghat, Solan 173234, Himachal Pradesh, India; 4 Department of Chemistry, Government Degree College, Una 174303, Himachal Pradesh, India; sunildutt.iitmandi@gmail.com; 5 Pranveer Singh Institute of Technology, Kanpur 209305, Uttar Pradesh, India; vivek.mishra@psit.ac.in; 6 School of Health Sciences and Technology, UPES University, Dehradun 248007, Uttarakhand, India

**Keywords:** Artificial Intelligence, wearable biosensors, biosensor, diabetes, cancer, COVID

## Abstract

Integrating artificial intelligence (AI) with wearable sensor technologies can revolutionize the monitoring and management of various chronic diseases and acute conditions. AI-integrated wearables are categorized by their underlying sensing techniques, such as electrochemical, colorimetric, chemical, optical, and pressure/stain. AI algorithms enhance the efficacy of wearable sensors by offering personalized, continuous supervision and predictive analysis, assisting in time recognition, and optimizing therapeutic modalities. This manuscript explores the recent advances and developments in AI-powered wearable sensing technologies and their use in the management of chronic diseases, including COVID-19, Diabetes, and Cancer. AI-based wearables for heart rate and heart rate variability, oxygen saturation, respiratory rate, and temperature sensors are reviewed for their potential in managing COVID-19. For Diabetes management, AI-based wearables, including continuous glucose monitoring sensors, AI-driven insulin pumps, and closed-loop systems, are reviewed. The role of AI-based wearables in biomarker tracking and analysis, thermal imaging, and ultrasound device-based sensing for cancer management is reviewed. Ultimately, this report also highlights the current challenges and future directions for developing and deploying AI-integrated wearable sensors with accuracy, scalability, and integration into clinical practice for these critical health conditions.

## 1. Introduction

Chronic conditions such as cancer, diabetes, and acute infectious diseases like COVID-19 place a tremendous burden on global healthcare systems. Cancer remains one of the leading causes of death globally, mainly due to late diagnosis and limited ability for continuous monitoring of disease progression and treatment response [1]. Conventional diagnostic methods, such as biopsies and imaging, often require multiple clinical visits and are invasive, costly, and time-consuming [2]. Diabetes, a chronic metabolic disorder characterized by persistent hyperglycemia, is another global health challenge. For effective management of diabetes, continuous monitoring of blood glucose levels is needed to avoid complications such as neuropathy, cardiovascular disease, and kidney failure. Traditional glucose monitoring methods, such as finger-prick tests, are invasive and provide only discrete data points [3]. The COVID-19 pandemic has further highlighted the importance of rapid, remote, and scalable diagnostics techniques. Traditional testing approaches, such as RT-PCR and antigen tests, although they are accurate, are limited by sample collection procedures, processing time, and logistical challenges during large outbreaks [4].

Conventional diagnosis approaches for cancer, diabetes, and COVID-19 often involve periodic visits to healthcare providers, submitting the sample for analysis, which can result in delayed detection of disease progression or complications. It is time-consuming, expensive, and requires healthcare experts for sample collection and analysis. Late diagnosis of these diseases significantly impacts the quality of life for patients. Moreover, there is a high chance of death due to the severity of the disease [5].

In recent years, healthcare systems globally have undergone a tremendous transformation, driven by rapid progress in technology, especially wearable sensing devices and Artificial Intelligence (AI). Wearable sensing technologies have emerged as an outstanding tool for continuous, non-invasive health monitoring. AI can quickly process vast amounts of data, analyzing it, recognizing patterns, comparing it with a reference dataset, and identifying health conditions [6]. Hence, AI has been widely used in various fields such as environmental monitoring, healthcare, food, automobiles, electronics, and sports [7]. AI integration in wearable sensing technology leads to the development of technology capable of managing cancer, diabetes, and COVID-19 [4,5,8,9].

In cancer, AI-based wearable biosensors are promising alternatives, enabling real-time detection of biomarkers associated with tumor growth, metabolic changes, and therapeutic efficacy. AI-based wearables enable early cancer diagnosis, continuous monitoring, and enhanced patient compliance. All these can lead to better clinical outcomes [4]. In the case of diabetes, AI-based wearable sensing technologies enable continuous glucose monitoring, offering real-time data on glucose trends. Further, they provide predictive analytics, personalized insulin dosing, and real-time alerts for hypo- or hyperglycemic events [8]. All these enable better glycemic control and reduce patient effort. In the case of COVID-19, AI-based wearable sensing devices emerged as a key technology in pandemic management. They allow continuous monitoring of physiological parameters such as heart rate, oxygen saturation, respiratory rate, and body temperature. By leveraging machine learning models, these devices can detect subtle changes in vital signs that often precede the appearance of COVID-19 symptoms, enabling early detection, remote monitoring, and timely medical interventions while reducing the burden on healthcare systems [10].

Recently, AI-based wearable sensing technologies have made significant progress in the field of healthcare [11,12,13]. The most recent achievements in the field of AI-based wearable sensing technologies include wearable photonic wristbands for cardiorespiratory function analysis and biometric identification [14]. It has potential for non-invasive and regular monitoring. Hybrid AI-based, bio-inspired wearable sensors with AquaSense AI technology are used in multimodal health monitoring and rehabilitation in dynamic environments [15]. Liu et al. [16] We utilized supervised machine learning technology to analyze data from 40 patients’ smartwatches. It demonstrated excellent predictive accuracy for mortality in end-of-life cancer patients, achieving 93%. In another study, Torrente et al. [17] Utilized datasets comprising wearable data from 350 patients and clinical data from 5275 patients, applying multilayer neural network technology for cancer prognosis, risk stratification, and follow-up management. The AI successfully constructed risk profiles of patients.

Despite their vast potential, AI-based wearable sensing devices still face challenges, including sensor accuracy, limitations, data privacy and security concerns, high development costs, interoperability issues, and the need for rigorous clinical validation. However, advancements in materials science, data analytics, cloud computing, and machine learning are rapidly addressing these challenges, paving the way for their integration into everyday clinical practice.

This review comprehensively explores the state-of-the-art advancements in AI-based wearable sensing technologies for the management of cancer, diabetes, and COVID-19. It discusses the various sensing modalities, the role of AI in data interpretation, predictive modeling, and decision support, as well as current challenges and future directions. This work aims to provide an understanding of how AI-based wearable diagnostics are poised to transfer disease management into a more precise, efficient, and patient-centered paradigm.

## 2. Artificial Intelligence-Based Wearable Sensing Techniques

Traditional diagnostic techniques are time-consuming, inaccurate, unable to detect disease early, not reproducible, and delay treatment significantly due to a vast number of patients waiting and limited infrastructure for diagnosis. All these may lead to patients being put in unnecessary circumstances. A lack of adequate, dependable, and cost-effective identification and real-time supervision restricts the affordability of early diagnosis and therapy [18,19]. The success of diagnosing a disease depends on the ability to detect the disease at an early stage, the accuracy of detection, real-time monitoring, and sensitivity and selectivity. Towards this direction, wearable sensing technology has made significant advancements.

The wearable sensing technology market is an emerging segment of the flexible electronics market, which was valued at $1.5 billion in 2020 and is expected to grow at an annual rate of 10.7% through 2030, significantly impacting global health in unprecedented ways. Wearables are small electronic devices designed to be worn on the body. Sensors used in wearable technology can be of various types, including electrochemical, colorimetric, optical, and pressure/strain sensors. These sensors can collect multiple types of signals, including physical, chemical, and biochemical signals. Patients can wear devices directly on their skin, driving rapid advancements in wearable technology for human health monitoring and tracking. Wearable sensing technology provides real-time physiological parameters through flexible, user-friendly devices. The results are faster, eliminating the need for a medical expert and enabling more rapid decision-making for timely treatment [20]. The significant challenges associated with wearable sensing technology are processing, analyzing, and predicting, in real time and continuously, the patterns of data collected by wearable sensors across a range of analytes. One of the best technologies for handling massive datasets is AI.

Artificial intelligence is the most strategic and disruptive technology available today. It marks the beginning of a new era of scientific, technological, and industrial revolutions, bringing powerful empowerment that significantly impacts social progress, economic growth, and the creation of the metaverse environment. Significant developments have been made across various areas, including environments, medicine, healthcare, agriculture, and industrial automation, through the application of artificial intelligence (AI) systems.

**Figure 1:**
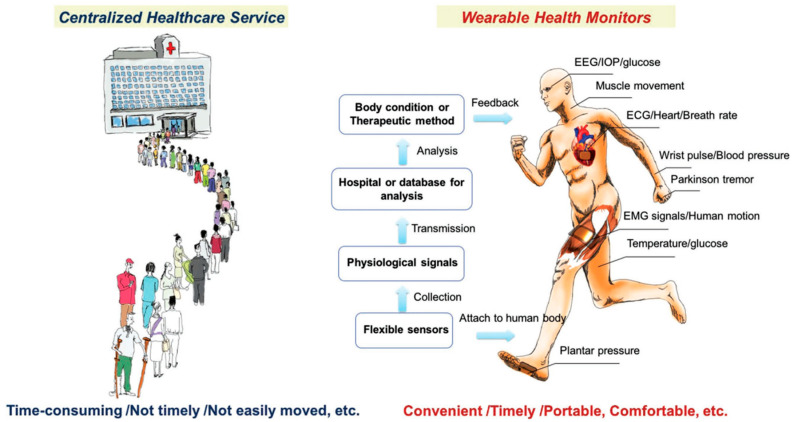
Illustration of differences between conventional healthcare services and wearable healthcare services. Reproduced with permission from ref. [21]. Copyright 2017 Wiley-VCH.

Integrating sensors and devices with AI empowers the development of AI in medical diagnosis and treatment, leveraging emerging sensing technologies. The significant advantages of wearable health sensing technologies over conventional healthcare services are depicted in Figure 1 [21]. Wearable devices can gather data, but machine learning (ML) algorithms address how this data can be used in sensing technology. The patterns derived from the collected data can be provided to healthcare providers to identify trends that can predict patient health outcomes. Suitable care can be provided based on the data analyzed [22]. Different diseases, such as Parkinson’s and multiple sclerosis, can be analyzed and treated using ML algorithms from a database [23]. Crucial information can be collected via these devices, which can facilitate considerable attention to continuous monitoring in non-clinical human health and fitness trials [21]. Saliva, sweat, and tears contain biomarkers found in body fluids, skin odor, breath, and pressure/strain monitoring, which can also be used in human wearable devices [24]. Heart rate, blood pressure, oxygen level, respiratory rate, and temperature are some signals that can be used in AI-powered wearable technology.

### 2.1. Artificial Intelligence-Based Wearable Electrochemical Sensing

**Figure 2:**
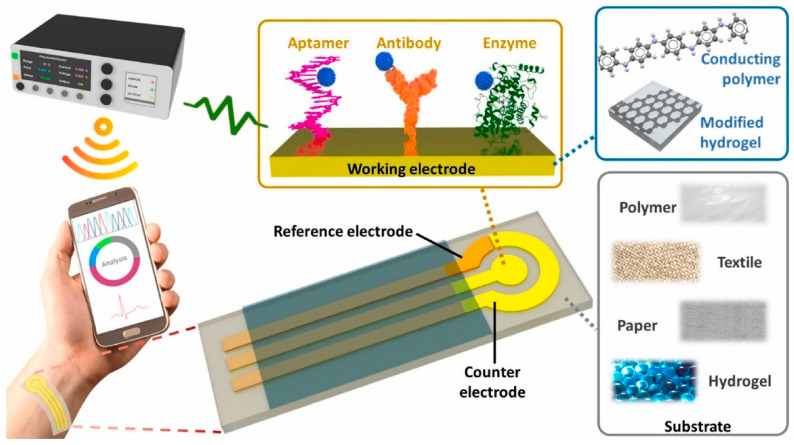
Schematic of wearable electrochemical biosensors with three electrode systems. Reproduced with permission from ref. [29]. Copyright 2024 Elsevier Open Access.

Electrochemical sensors detect analytes by translating biochemical interactions into electrical signals through mechanisms such as amperometry, potentiometry, or conductometry [25]. Electrochemical sensing techniques are widely used for glucose and lactate detection, as well as for biomarkers in sweat, saliva, or interstitial fluid. It further offers minimally invasive and continuous health monitoring [26]. Selectivity among potential biologically interacting chemicals and background noise in clinical samples are two significant biosensor problems that are unsolvable by traditional biosensing techniques. It remains unclear how embedded machine learning can be used to eliminate background interference, which is frequently present in complex matrices such as cerebrospinal fluid. Machine learning can be applied to sensors and devices to improve their ability to distinguish between different responses. Furthermore, incorporating these models into wearable electronics can enhance the use of point-of-care tools, ongoing surveillance, and evaluations of viral mutations [27]. Body fluids often have lower concentrations of biological analytes than blood. Therefore, several biosensor characteristics, including sensitivity, limit of detection, selectivity, and reaction time, must be optimized for consistent, reliable wearable monitoring. Smaller electronics and flexible materials are also necessary for portable point-of-care wearable designs [28]. The fundamental components of all body-worn electrochemical biosensors comprise various components such as (i) a substrate that maintains direct touch with the human body, (ii) electrochemical three-electrode systems compose of working (WE), counter (CE), and reference (RE) electrodes, (iii) immobilized bio-receptors or recognition elements that preferentially interact with the complementary analyte to cause a biochemical shift on the working electrode. The bio-signal is converted into an electrical signal by the transducer, which is then captured, processed, and displayed by (iv) amplifier circuits, (v) electrochemical analyzers, (vi) wireless communication modules, and (vii) software and displayers. The schematic of the wearable electrochemical sensing platform is depicted in Figure 2 [29].

Sweat, urine, saliva, and tears are electrochemical sensing biomarkers for health monitoring, disease diagnosis, and health monitoring [30]. Impedance, current, potential, and conductance are the electrical signals that an electrochemical sensor generates due to the binding of a bioreceptor with a specific analyte [31]. Electrochemical wearable sensors are extensively utilized owing to their high performance, affordability, small size, and broad range of applications [32]. Electrochemical sensors developed using various nanomaterials, such as metal–organic frameworks (MOFs) [33], transition metal dichalcogenides [34], carbon nanomaterials [35,36], metal-oxides [37], and conducting polymers [38]. It delivers better performance than conventional electrochemical sensors. Electrochemical sensors use enzymes, antibodies, or other molecules as analytes. The barely detectable amount of several metabolites and minerals, including all necessary vitamins and amino acids, was measured using the NutriTrek electrochemical sensors. This biosensor is made up of graphene electrodes. Metabolite-targeted antibody-mimicking molecularly imprinted polymers and redox-sensitive nanoparticles applied for the electrode’s functionalization [32].

Sensors for wearable technology that detect biomarkers at trace levels are adaptable to living tissues and physiological movements, enabling accurate tracking of these biomarkers. Several innovative modification techniques have been devised to increase the sensitivity and flexibility of wearables. Recently, hydrogels have been used extensively as electrode modifiers in wearables owing to their outstanding stretchability and adaptability with human soft tissues [39]. Hydrogel-based wearable electrochemical biosensors do not damage living tissues, enabling extended supervision and high accuracy in identifying the desired biomarkers.

### 2.2. Artificial Intelligence-Based Wearable Colorimetric Sensing

**Figure 3:**
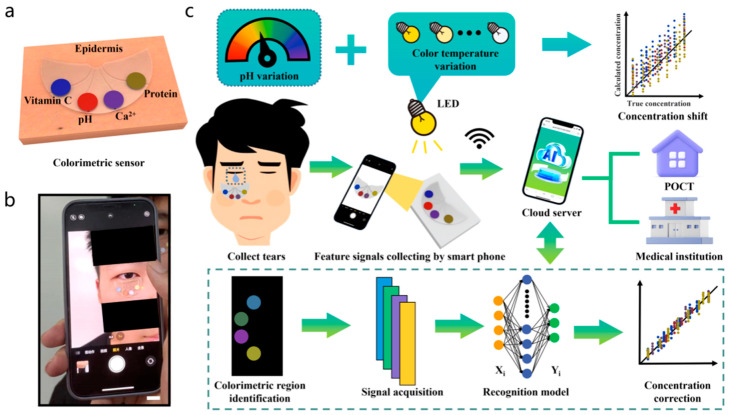
AI-based wearable microfluidic colorimetric sensing technology. (**a**) microfluidic colorimetric sensing, (**b**) collection of color data by smartphone camera, and (**c**) colorimetric sensing of biomarkers in human tears using AI. Reproduced with permission from ref. [42]. Copyright 2024 Springer Nature Open Access.

Colorimetric sensing is a robust, straightforward diagnostic technique that relies on visually perceptible color changes in response to physiological and biochemical stimuli. It is generally applied in paper-based test strips and patches. By integrating colorimetric sensing techniques into skin patches, textiles, and contact lenses, real-time monitoring of sweat, saliva, and tears is enabled [40]. Microfluidic colorimetric tear sensors visually represent variations in tear biomarker concentration by colorimetric reactions, in contrast to electrochemical tear sensors. It is crucial to precisely, simultaneously, and quickly identify biomarkers in human tears to monitor eye and overall body health. However, there are difficulties with the colorimetric sensor’s data gathering, interpretation, and sharing, which limit the technology’s usefulness [41]. To overcome the challenges of conventional colorimetric sensing technology, an AI-based cloud server data analysis system (CSDAS) integrated into an electronic device, such as a smartphone, provides color data capture, evaluation, self-adjustment, and visualization. Similarly, Wang et al. developed an AI-assisted wearable microfluidic colorimetric sensor (AI-WMCS). The working mechanism is depicted in Figure 3. Briefly, AI-WMCS is composed of a PDMS microfluidic patch that collects tears and directs them to a channel reservoir containing paper mounted with a chromogenic reagent. Using smartphones, they have captured the data and uploaded it to CSDAS, which quickly identifies colorimetric regions. Based on the results, it detects targeted biomarkers [42]. Further, they have built a CNN-GRU neural network model dataset through collecting a large number of color data for each concentration of biomarkers at different temperatures and pH. The self-learning process builds a calibration curve, thereby further boosting sensor sensitivity. It also helps avoid errors caused by changes in temperature or pH. Hence, such wearable sensing technology will be grateful for monitoring various analytes in tear samples with high sensitivity and selectivity, without error, at different temperatures and pH levels.

Paper-based analytical devices (PADs) that use colorimetric sensing and mobile photos have become increasingly popular in various sensing applications [43]. A combination of three image color spaces, RGB, HSV, and LAB, and four machine learning (ML) models: logistic regression, support vector machine (SVM), random forest, and artificial neural network (ANN) for their precision in predicting analyte concentrations. It is also further demonstrated that using the right models and color spaces together can be a powerful, quick, and low-cost method for field testing on many samples on colorimetric PADs. Cui et al. also reported an AI-assisted colorimetric biosensor for smartphones that detects harmful germs quickly and accurately in a visible manner [44]. Hyaluronic acid (HA) hydrogel loaded with β-galactosidase (β-gal) and loaded with β-D-galactopyranoside (CPRG) serves as the bioreactor and signal generator in the biosensor. Gram-positive and Gram-negative bacteria can be recognized by the biosensor, which can produce a report in less than 60 min with an ultra-low LoD of 10 CFU/mL [44].

Colorimetric tear-based sensors face challenges in reproducibility and precision. However, it can achieve quantitative accuracy by using standard color reference charts and digital color calibration, enabling consistent mapping of color intensity to accurately detect analyte concentration. Image processing algorithms integrated with smartphones and spectrometers reduce human error in color interpretation. Machine learning can enhance detection sensitivity by mitigating lighting variability, user handling, and background interference. Microfluidic channels equipped with advanced tear sensors may enable precise control over sample volume and reaction kinetics, thereby enhancing reproducibility. Quantitative tear sensor validation using gold-standard clinical assays can ensure accuracy in real-world use.

### 2.3. Artificial Intelligence-Based Wearable Chemical Sensing

Chemical sensing involves detecting target analytes, such as electrolytes, metabolites, and gases, in biofluids like sweat, saliva, and interstitial fluid using selective chemical recognition elements. It offers a physiological status and disease progression [45]. Chemical sensing based on nanomaterials is among the most popular sensors for analyte detection. Conductive inorganic nanomaterials, such as metal and carbon nanomaterials conjugated with ligands or functional groups, are commonly used in construction applications for sensors. Chemiresistor sensing behavior is influenced by two main mechanisms: (a) a three-dimensional swelling and/or aggregation of the film, which alters electrical resistance by changing the interparticle tunneling distance for charge transport to/from the conductive core upon analyte exposure; (b) a boost in the organic matrix’s permittivity, which decreases potential barriers between organic cores [46,47]. One challenge in creating wearables with chemical sensors for health monitoring is detecting and quantifying the targeted analyte. For example, ammonia (NH_3_) is present at 0.5–2 ppm in human exhaled breath.

**Figure 4:**
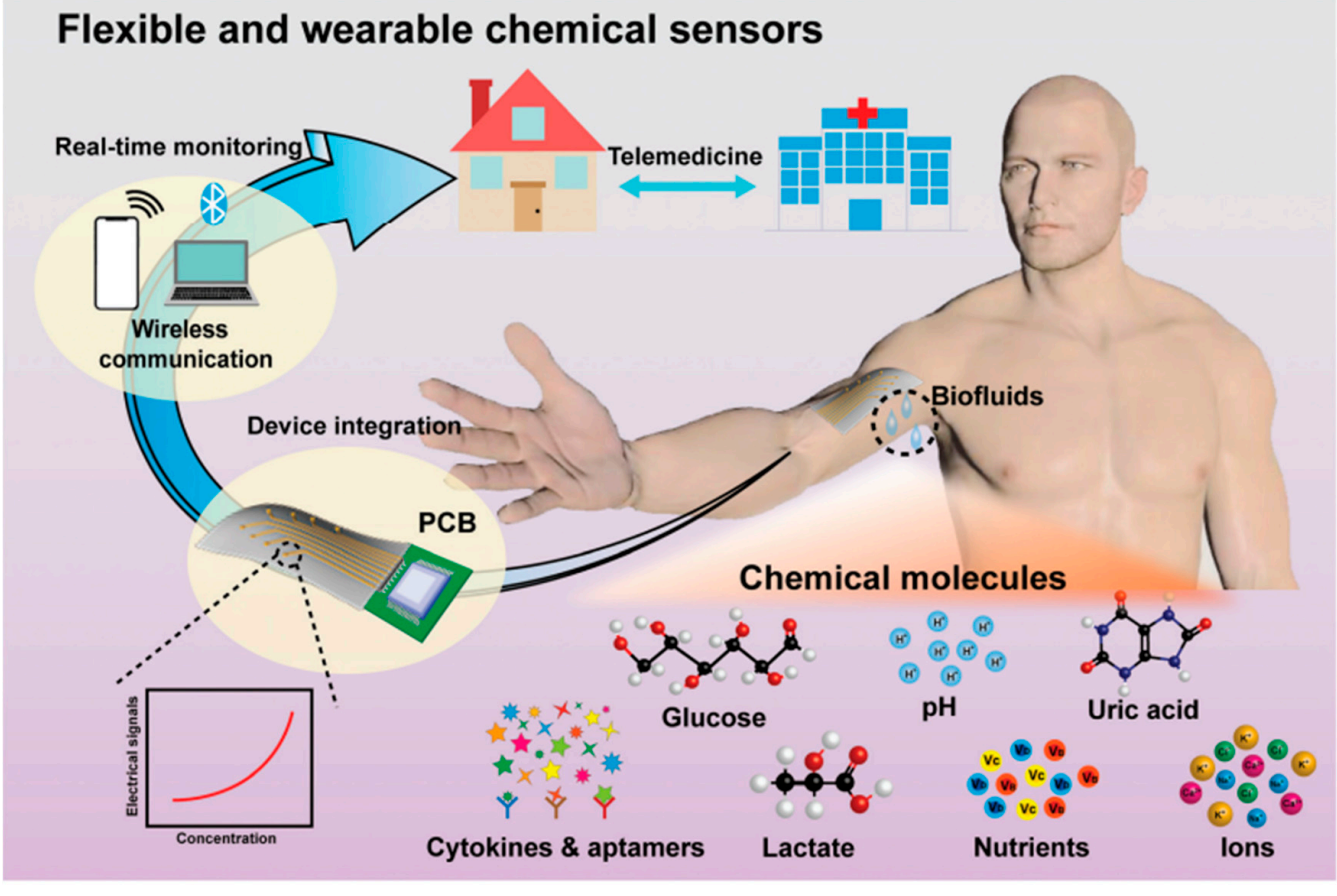
AI-based wearable chemical sensors. Reproduced with permission from ref. [49]. Copyright 2022 The Royal Society of Chemistry.

The concentration of biomarkers is also crucial to monitor for diagnosis. A higher concentration of biomarkers may suggest kidney or liver problems, whereas a lower concentration may suggest asthma symptoms [48]. Furthermore, not every disease has a known biomarker. Therefore, appropriate ML algorithms are being used to enhance the effectiveness of sensors in evaluating large volumes of acquired data, thereby sending timely notifications to achieve the central purpose of smart health supervising technology. The schematic of AI-based wearable chemical sensors for the detection of chemical molecules such as pH levels, glucose, lactate, uric acid, ion levels, cytokines, nutrients, and other biomarkers is depicted in Figure 4 [49].

### 2.4. Artificial Intelligence-Based Wearable Optical Sensing

Optical sensing techniques rely on light–matter interactions, such as absorption, reflection, fluorescence, or scattering, to non-invasively analyze physiological parameters, including heart rate, oxygen saturation, glucose levels, and tissue perfusion [50]. Optical sensors are often integrated into smart watches, patches, and textile devices. Usually, they are user-friendly health monitoring devices. In recent decades, human beings have witnessed the profound impact of photoelectronic sensing technology on all aspects of human civilization, including massive data, cloud servers, closed-loop systems, intelligent IoT gateways, AI, blockchain, and metaverse. Functional optoelectronic devices made from various materials, including semiconductors, are a crucial component of the AI optoelectronic sensing method. A necessary part of electronic systems, the optoelectronic display (a display) projects text, pictures, and video data for easily understood visualization that supports human cognition. The use of wearable optical smart devices for real-time biomarker detection is growing. Future wearable technologies will rely heavily on optics and photonics to perform highly sensitive measurements of factors that would otherwise go undetected, providing valuable information about our environment and health. The increasing use of optical wearable technologies, such as glucose, blood pressure, and heart rate monitors, makes personalized medicine a reality by allowing users to create complex, multidimensional physiological and environmental data [51]. The functions of different kinds of optoelectronic sensors are often closely tied to any breakthroughs in AI optoelectronic sensing technology. Artificial Intelligence (AI) is concerned with finding solutions to problems involving machines that can see (word and image recognition), hear (machine translation, speech recognition, etc.), think (human–machine interface), and speech (human–machine communication, speech synthesis, etc.). These are the most pressing issues where AI is currently being integrated. Optoelectronic sensing technology is often used to address these issues. As the AI era develops, optoelectronic wearables are being incorporated into an increasing number of gadgets and products. Compact, digital, and intelligent sensors are becoming increasingly common and are revolutionizing our way of life. Photoelectric sensors, for example, translate an optical signal into an electrical signal.

Machine translation, speech recognition, picture and word recognition, and human–machine communication are the top challenges AI is currently addressing, and AI is focused on tackling these issues. An AI-based Optoelectronic detection system is frequently used to address these issues. AI optoelectronic sensing technology relies heavily on functional optoelectronic devices, which are made of a range of materials such as semiconductors, organic optoelectronic materials, and 2D materials [52]. The role of different kinds of optoelectronic sensors is often closely tied to advancements in AI optoelectronic sensing technology. The optoelectronic effect results from light shining on certain materials; electrons convert photon energy, triggering an electrical response [53].

### 2.5. Artificial Intelligence-Based Wearable Pressure/Strain Sensing

**Figure 5:**
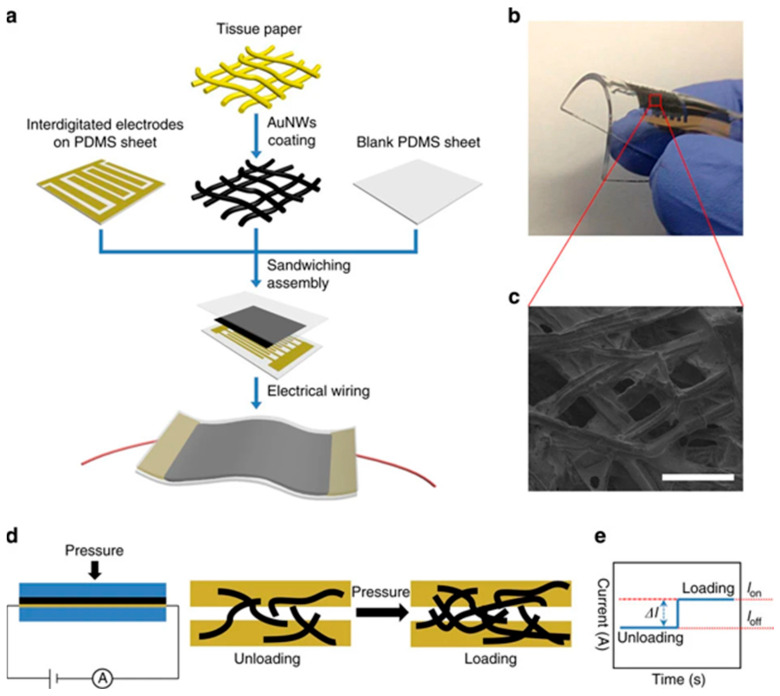
The pressure sensor is based on the AuNW-coated tissue paper. (**a**) flexible sensor fabrication procedure, its (**b**) photograph exhibiting bendability, (**c**) SEM of AuNWs coated tissue fibers, (**d**) sensing mechanism, and (**e**) changes in current due to loading and unloading. Reproduced with permission from ref. [56]. Copyright 2024 Macmillan Open Access.

Due to their numerous applications in industrial monitoring and personal electronic devices, pressure sensors are appealing options for advancing AI-based wearable sensing technologies. Organic-material-based flexible pressure sensors, which offer the extraordinary benefits of affordability and flexibility, are a rapidly growing field due to their potential applications in wearable sensing technology and artificial intelligence systems. Large-scale movements, such as the motion of human joints, and small-scale pressures and motions, such as a faint touch or heartbeat, are detected by electromechanical sensors. Consequently, they fall within the category of strain and pressure sensors. Under desired force, pressure sensors can generate signals and act via a process known as signal transduction. This key characteristic enables pressure sensors to be successfully used in industrial production, artificial intelligence, and personal electronic devices [54,55]. Recently, the wearable, precise pressure sensor developed using ultrathin AuNW-coated tissue paper is depicted in Figure 5 [56]. They first fabricated AuNWs and coated them on Kimberly-Clark tissue through a dip-coating and drying approach. Then, the AuNW-coated tissue paper is sandwiched between a PDMS sheet and a PDMS-integrated electrode. The SEM image showed uniform deposition of AuNWs. The fabricated device is wearable and bendable. Then they fabricated pressure sensors that change current with loading or unloading. Strain sensors measure changes in capacitance or resistance to transform mechanical deformations into electrical signals [57,58]. Essential parts of e-skins, exceptionally responsive, adaptable, and stretchy pressure detectors, are also considered for wearable health-tracking devices [59]. Pressure sensors have various applications, including hearing aids, e-skin, daily activities, and measuring weight and height.

Pressure sensors operate via different mechanisms, such as transduction, and there are several methods for converting tactile impulses into electrical signals, including piezo resistance, capacitance, and piezoelectricity [60,61]. Studying artificial intelligence, or human-like intelligence exhibited by computers and software, is crucial for the development of flexible pressure sensors [62]. Artificial intelligence technology has advanced significantly over the last decade. In interactive robots that utilize AI, the high-performance integration of compliant pressure-sensing devices is required. Blood pressure and pulse are continuously recorded to gather basic health data using highly pressure-sensitive sensors [63]. Monitoring blood pressure, heart rate, and pulse is essential for maintaining human health. Wearable smart bracelets have been widely used in recent years to monitor health in real time, attracting considerable attention. A carbon nanotube (CNT)–polydimethylsiloxane (PDMS) composite is used to fabricate flexible, biocompatible dry electrodes for long, high-performance wearable electrocardiographic (ECG) monitoring devices [64].

## 3. Artificial Intelligence-Based Wearable Sensing Technology for COVID-19 Management

**Figure 6:**
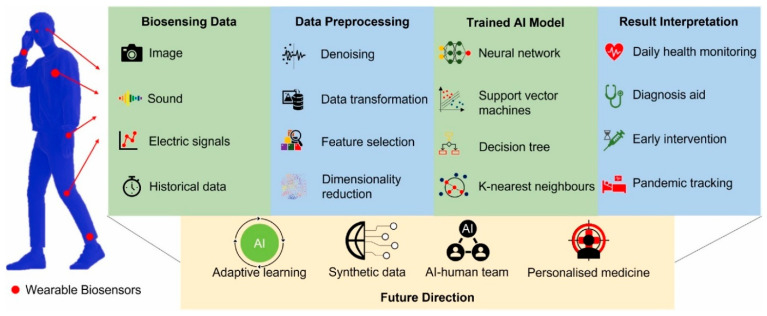
Current Applications and future research directions of AI algorithms in the biosensing field. Reproduced with permission from ref. [67]. Copyright 2022 Elsevier Open Access.

The COVID-19 pandemic, caused by the SARS-CoV-2 virus, has significantly transformed healthcare systems worldwide, underscoring the need to adopt various cutting-edge technologies to manage infectious diseases. The rapid spread of the virus and the emergence of its several mutations have made it difficult to diagnose the infection in its early stages and to track patients over time [65]. Conventional healthcare approaches, like hospital-based treatment and diagnostic tests, have not met the volume and urgency of prompt intervention needs. As a result, Artificial Intelligence and wearable sensing technologies have become essential tools for managing and monitoring various health conditions, including pandemics, in real-time [66]. Figure 6 depicts AI in biosensing data collection, processing, training algorithms, and result interpretation, as well as AI applications in future research directions, such as adaptive learning, synthetic data, AI–human team, and personalized medicine [67].

Wearable health monitoring devices, notably smartwatches and intelligent clothing, can track vital physiological indicators like heart rate, body temperature, and oxygen saturation (SpO_2_), making them a prominent and indispensable part of the common man’s daily life [68]. When coupled with AI, these devices provide a more dynamic strategy for predictive diagnostics, remote health management, and symptom detection [69]. By evaluating massive amounts of data in real-time, providing individualized health insights, and identifying anomalies early, Artificial Intelligence (AI) significantly improves the effectiveness of wearable sensing systems. In the subsequent subsections, various AI-based wearable device research studies on COVID-19 management have been classified by sensing technology and relevant health parameters to facilitate an in-depth review.

### 3.1. Heart Rate and Heart Rate Variability (HRV) Sensors

Heart rate (HR) and heart rate variability (HRV) sensors have been essential in detecting and treating COVID-19. They have enabled real-time, non-invasive monitoring of cardiovascular parameters by measuring myocardial damage. Since the onset of COVID-19, clinical research has shown that the virus affects the cardiovascular and respiratory systems, and alterations in HR and HRV are frequently the first indicators of infection-related physiological stress. Due to this, wearable technology has emerged as a vital and affordable tool for real-time HR monitoring in COVID-19 patients. Using wearable technology, including smartwatches and chest patches with photoplethysmography (PPG) sensors to measure patients’ vital signs continually, numerous studies have investigated the usefulness of these metrics in diagnosing and predicting COVID-19 infection [70]. Preliminary research on early-stage COVID-19 infection indicates that patients often exhibit a higher resting heart rate (RHR) and reduced heart rate variability (HRV) due to dysregulation of the autonomic nervous system (ANS). Wearable technologies, such as the Apple Watch and Oura Ring, can continuously monitor minute variations in HR and HRV, enabling detection of deviations even before the onset of clinical symptoms, as interpreted by Chatterjee et al. [71]. Wearable devices equipped with piezoresistive sensors that provide real-time, high-accuracy heart rate monitoring are a suitable choice for managing COVID-19 patients. The potential of wearable devices in pre-symptomatic screening was highlighted by Mishra et al. [70], who demonstrated that monitoring changes in resting heart rate and heart rate variability may predict COVID-19 onset up to 9 days before symptoms. They used RHR-Diff and HROS-AD algorithms to monitor RHR, steps, and sleep duration using a smartwatch. They processed 5262 participants, including 32 COVID-positive individuals. This system detected 81% of COVID cases and 88% of cases before the symptoms. The real-time CuSum algorithm exhibited 63% sensitivity. Similar results were observed by Quer et al. [50], who discovered that the accuracy of early COVID-19 detection was significantly increased when HRV and temperature data from wearables were combined [72]. Shahshahani et al. [73], emphasize the accurate positioning of the sensors and created a wearable with an ultrasonic sensor for monitoring heart rate and ECG signals for providing care to COVID-19 patients. Using the Internet of Things, Bhardwaj et al. developed a health monitoring system that combines temperature, SpO_2_, and heartbeat sensors for remote COVID-19 patient care [74]. Additionally, researchers has demonstrated that HRV can predict illness severity, with lower HRV associated with worse clinical outcomes [68].

AI has improved early detection and predictive modeling capabilities by integrating with HR and HRV sensors. To identify patterns indicative of COVID-19 infection, machine learning techniques, such as neural networks, have been used to analyze HR and HRV data in real-time [66]. According to a study by Riaz et al., AI models evaluating HRV data from wearables identified probable COVID-19 instances with high accuracy [69]. Public health monitoring systems are increasingly integrating AI-driven algorithms that enable scalable, effective identification of COVID-19 in asymptomatic individuals, as reported by Hasasneh et al. [75]. Furthermore, as patients with prolonged COVID-19 frequently experience extended autonomic dysfunction, which can be monitored with wearable technology, continuous monitoring of HR and HRV metrics has been beneficial for managing these patients [76].

### 3.2. Oxygen Saturation (SpO_2_) Sensors

Furthermore, oxygen saturation (SpO_2_) sensors have become a vital tool in managing respiratory disorders, particularly during the COVID-19 pandemic, as they can non-invasively measure blood oxygen levels by quantifying the fraction of oxygen-saturated hemoglobin. SpO_2_ levels typically range from 95% to 100% in healthy people [77]. Further studies confirmed that patients with severe COVID-19 often have significantly lower resting SpO_2_ levels, indicating greater respiratory compromise. Pulse oximeters are the prominent devices for SpO_2_ monitoring and have become crucial during the pandemic in detecting “silent hypoxia”. It is a condition in which individuals show dangerously low oxygen levels without exhibiting symptoms [78]. Further, Costrada et al. suggested combining continuous SpO_2_ monitoring with other critical metrics, such as body temperature, to design a compact, low-power wearable SpO_2_-measuring device [79]. This makes continuous monitoring more feasible, particularly for home-based care. Phillip et al.’s wearable system combines heart rate variability (HRV) and SpO_2_ tracking, ensuring precision using a commercial wrist-worn pulse oximeter [80]. This device monitors COVID-19 patients recovering at home, identifies significant reductions in SpO_2_, and treats them immediately. Furthermore, the accuracy and adaptability of SpO_2_ monitoring have improved significantly with the incorporation of cutting-edge wearables, such as the Oxitone 1000M. With an error rate of less than 3% for SpO_2_ measurement, the Oxitone 1000M is the first FDA-approved wrist sensor pulse oximetry monitor in history [81]. This gadget offers patients with diverse demands flexibility because it can be worn on different regions of the body, such as the head and chest. These advancements are crucial for enhancing patient comfort and adherence, particularly in situations that require long-term monitoring, such as post-COVID-19 recovery management.

**Figure 7:**
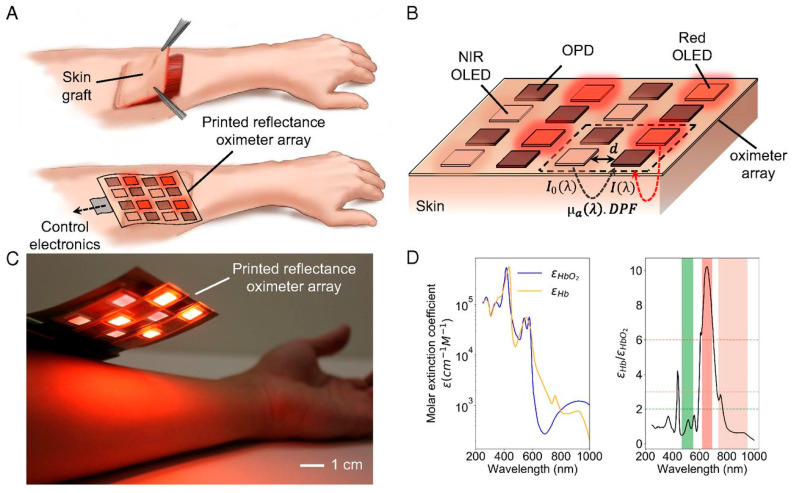
Illustration and mechanism of the printed reflectance oximeter array (ROA). (**A**) ROA using process, (**B**) ROA configuration, (**C**) picture of ROA over a person’s forearm, and (**D**) the molar extinction coefficients of HbO_2_, Hb, and their ratio. Reproduced with permission from ref. [82]. Copyright 2018 PNAS Open Access.

Painless oxygenation mapping efficacy plays a crucial role in post-surgery monitoring of wounds, tissues, and organs. Khan et al. reported A flexible organic reflectance oximeter array (ROA). The schematic and working mechanism are depicted in Figure 7. Briefly, the ROA sensor comprises a Red and NIR OLED array arranged in a checkerboard pattern. The OPD array is placed over the OLED array. OLED acts as a light emitter, and OPD collects diffused reflected light. By placing the ROA on a skin graft after surgery, it can perform oxygenation mapping. The absorption coefficients depend on the specific absorption coefficient and concentration of HbO_2_, Hb, and DPF, which are the differential pathlength factors [82]. A device for monitoring blood oxygen saturation (SpO_2_) was designed using organic light-emitting diodes (OLEDs) and organic photodiodes (OPDs), integrated into organic photoplethysmography (PPG) biosensor systems fabricated on flexible polymeric substrates. This device provides efficient, conformal human–machine interfaces that enable long-term monitoring of biochemical signals. Healthcare professionals can now remotely monitor patients using this device’s compact size, easily measurable features, and support for the Internet of Things (IoT). Similarly, Sarkar and Assaad [83]. Utilize photoplethysmography (PPG) and light spectroscopy to design a reflective mode polarized imaging-based PPG to measure non-contact heart rate and SpO_2_. They customized an image sensor with wire-grid polarizers that detects phase information from backscattered light.

### 3.3. Temperature Sensors

The frequent presentation of fever as a primary sign of COVID-19 has made temperature sensing indispensable for its management. Real-time body temperature monitoring has been made possible by wearable, non-contact temperature sensors, such as thermistors and infrared (IR) sensors. These sensors provide a non-invasive method of early fever detection. In public places like airports and hospitals, infrared-based detectors that detect radiation emitted by the human body are frequently used for screening people; however, their accuracy can be affected by temperature and proximity to the skin [84]. New developments in infrared (IR) and IoT-based wearables, such as those created by Ahmed et al. [85], combine cloud technologies with continuous temperature monitoring to enhance real-time data availability and healthcare response. Wearable sensors based on thermistors have become increasingly common in remote healthcare settings. Devices like the iThermonitor have continuously monitored fever in COVID-19 patients to facilitate remote patient care. These devices broadcast real-time data to telemedicine systems [86]. Flexible thermistor sensors have also been incorporated into intelligent textiles to improve patient comfort during long-term monitoring [87]. Known for their remarkable mechanical and electrical capabilities, graphene-based conductive materials are being integrated into flexible sensors. The latest developments in graphene-based flexible sensors emphasize their electrical and thermal conductivity, tensile strength, and novel sensing techniques for temperature, gas, and strain detection [88]. Additionally, it investigates novel fabrication techniques that improve sensor performance. These devices use thin, flexible materials that mold to the skin to continuously capture data [89].

**Figure 8:**
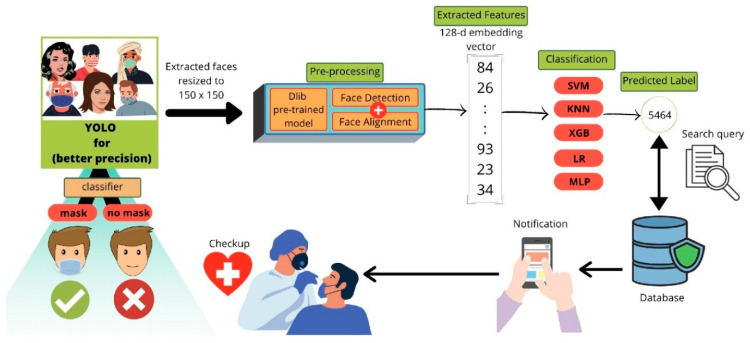
The flow chart of face mask identification and COVID-19 notification. Reproduced with permission from ref. [92]. Copyright 2021 Elsevier.

RFID-based temperature sensors have become valuable for non-contact monitoring in hospital settings. By integrating RFID with hospital management systems, they reduce the risk of cross-infection by remotely collecting and transferring temperature data for multiple COVID-19 patients [90]. AI integration has substantially improved the accuracy and usefulness of temperature sensors. The goal of the research conducted by Al-Humairi et al. is to create an artificial intelligence-powered smart helmet that can adapt to changing conditions, using thermal (Adafruit) and Pi (speed sensor) module cameras with embedded sensors [91]. It focuses on facial detection and real-time body temperature, both of which are calibrated using AI algorithms. These aspects are integrated into the system to provide adequate health monitoring. To identify potential COVID-19 cases in large crowds, Barnawi et al. developed an IoT-UAV-based system that analyzes thermal images using onboard thermal sensors. The thermal images recorded by the thermal camera were used to identify potential people in the pictures who may have COVID-19 based on the temperature recorded. Further, they integrated a face mask detection approach to determine whether a person is wearing a face mask. The workflow schematic of the designed COVID-19 notification system using face recognition is depicted in Figure 8. The schemes’ performance evaluation is performed using various machine learning and deep learning classifiers. It achieved an average accuracy of 99.5% across different performance metrics, strengthening its practical implications [92]. It employs a hybrid face recognition technique to detect and identify people with elevated body temperatures in real time. This technique improves the effectiveness of crowd monitoring for detecting COVID-19. The investigation conducted by Khaloufi et al. uses deep learning and smartphone-embedded MEMS (Micro-Electro-Mechanical Systems) sensors to make a preliminary COVID-19 diagnosis [93]. The proposed system extracts health-related features for early disease detection using a cutting-edge deep learning framework. It provides an easy-to-integrate, deployable smartphone screening solution that enables effective monitoring.

### 3.4. Respiratory Rate Sensors

Respiration rate sensors have become essential for combating COVID-19, as the virus has severely affected the respiratory system. Monitoring is vital to preventing the development of severe complications like acute respiratory distress syndrome (ARDS) [94]. Abnormally high respiratory rates are a precondition for respiratory distress in patients. As a result, numerous technical developments have been made to ensure precise and instantaneous monitoring of respiration rates [95]. Wearable devices are among the most commonly used technologies for respiratory rate monitoring, particularly for COVID-19 patients who require continuous monitoring. A study by Troyee et al. designed a wearable device that measures respiratory rate with great precision using piezoelectric sensors [96]. Logistic regression is also used to perform binary classification between people with respiratory problems and those without. This system is ideal for real-time monitoring in clinical and residential settings. Wearable breast bands and chest bands have also been incorporated with fiber-optic sensors. The highly sensitive monitoring of chest wall movements, made possible by fiber optics, enables the early detection of respiratory failure [97].

**Figure 9:**
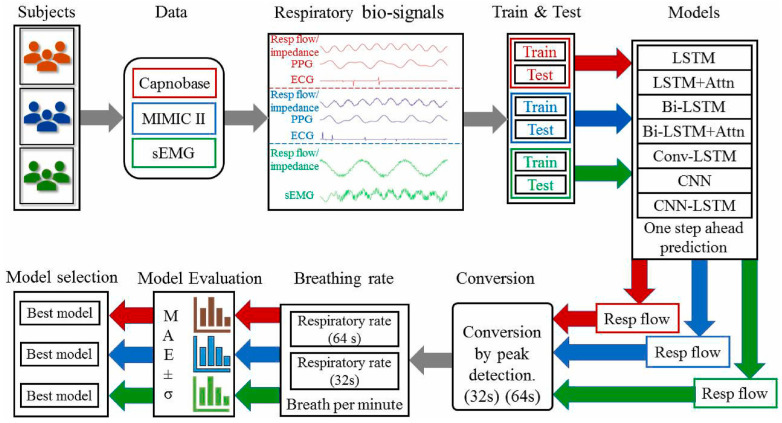
Flowchart of respiratory rate evaluation and prediction. Reproduced with permission from ref. [100]. Copyright 2022 Elsevier.

To facilitate caregivers with reduced risk of infection, a contactless device using infrared thermography has been designed by Jha et al. for respiration rate monitoring that can identify temperature changes associated with breathing [98]. This approach is highly accurate in controlled settings; however, external factors, such as ambient temperature, can affect its accuracy. Furthermore, radar-based technologies, such as the one proposed by Purnomo et al., use frequency-modulated continuous wave (FMCW) radar to track respiratory movements. This provides a non-invasive method of detecting minute chest motions, even under challenging circumstances [99]. Data analytics, machine learning, and artificial intelligence have improved respiratory rate monitoring. Kumar et al. presented convolutional neural networks (CNNs), a deep learning model, to interpret respiratory sensor data and enhance the detection of abnormal breathing patterns associated with COVID-19. The step-by-step process of detection and processing of the respiratory rate is depicted in Figure 9 [100]. They used data collected from contact-based sensors, ECG, PPG, and sEMG from different samples. ECG and PPG signals are vascular signals collected from blood vessel activity, whereas sEMG signals are collected from muscle activity. Datasets Capnobase and MIMIC-II include respiratory flow signals alongside PPG and ECG signals. In contrast, the sEMG dataset includes respiratory flow data alongside EMG during breathing and resting periods. The framework used PPG, ECG, respiratory flow, and EMG signals. The data is then split into test and training sets. A training set helps develop a model, whereas a test set helps evaluate it. They used deep learning models such as LSTM, LSTM + Attn, Bi-LSTM, Bi-LSTM + Attn, ConvLSTM, CNN, and CNN-LSTM. LSTM exhibited the best for one of the datasets, and Bi + LSTM showed the best for 2 of 3 datasets [100].

The study conducted by Cannata et al. for respiration rate monitoring classifies X-ray images using sophisticated artificial intelligence algorithms to diagnose COVID-19 [101]. It utilizes the Vision Transformer and transfer learning on pre-trained convolutional neural networks (InceptionV3, ResNet50, Xception) to reduce computational complexity and enhance detection accuracy. This approach provides an automated, effective way to identify COVID-19 infections. Rohmetra et al. stated that these cutting-edge AI algorithms for data analytics ensure that medical professionals can react promptly to the first indications of respiratory trouble [102].

Telemedicine platforms incorporated with respiratory rate sensors have become prominent for remote patient monitoring. Palanisamy et al. have presented a cloud-based remote platform for monitoring respiration rate that collects data from wearable sensors. These systems utilize Bluetooth- and Zigbee-capable devices that transmit real-time data to the cloud, where machine learning algorithms analyze it to identify indicators of respiratory distress. This technology has decreased in-person visits, and COVID-19 patients receive continuous care [103]. Furthermore, respiratory rate sensors are integrated into commercial devices like Fitbit and Apple Watch to provide at-home monitoring [104]. These devices are valuable tools for individuals with mild to moderate COVID-19 symptoms. They offer basic respiratory monitoring capabilities outside clinical settings, though they primarily rely on photoplethysmography (PPG) to estimate respiratory rate. They are also widely available and easy to use.

### 3.5. Multi-Sensor Devices

AI-enhanced multi-sensor devices that provide continuous tracking and early symptom detection play a significant role in managing COVID-19. These devices combine data from several sensors, including temperature, heart rate, SpO_2_, and respiration rate [105]. AI algorithms use data to identify early infection symptoms and predict disease severity. The artificial intelligence (AI) models improve COVID-19 diagnosis accuracy and reliability by integrating physiological data from multiple sources, minimizing the likelihood of false negative results [106]. Wearable devices that leverage AI-based multi-sensor systems for real-time monitoring are among the significant advancements in this field. The use of AI models in conjunction with multi-sensor wearables that incorporate temperature, heart rate, and SpO_2_ sensors to efficiently anticipate COVID-19 symptoms before they worsen [107]. Similarly, Shanker et al. evaluated temperature and breathing rate data in wearable multi-sensor systems using deep learning networks to identify anomalies early in COVID-19 patients [108]. Various AI-based wearable sensing devices for COVID management and their features, including type of wearable device, type of AI, target analyte, limit of detection, detection range, selectivity, specificity, pros and cons, are summarized in Table 1.

AI is also essential for identifying “silent hypoxia”, a crucial sign in severe COVID-19 cases. Multi-sensor devices that combine infrared and photoplethysmography (PPG) can track heart rate and oxygen saturation. These readings are analyzed by AI algorithms, which then produce real-time notifications for medical intervention. Furthermore, Yi et al. provided evidence that cloud-based AI systems can gather information from wearable multi-sensor devices across demographics, facilitating effective large-scale COVID-19 monitoring [109]. AI-driven multi-sensor systems help manage long-term COVID and identify active infections. Zimmerling and Chen tracked the virus’s long-term impacts and provided individualized insights on patient recovery by integrating respiratory data and heart rate variability (HRV) into AI algorithms [110]. Despite the enormous potential of multi-sensor systems, several key issues, including interoperability, noise, and sensor accuracy, remain under investigation.

**Table 1:** Summary of literature related to various AI-based wearable sensing technologies for COVID-19 management.

Type of Wearable Sensor	AI/Algorithm Used	Target Analyte (Intended)	Limit of Detection (LOD)	Detection Range	Selectivity	Specificity (Reported)	Pros	Cons	Ref.
Smartwatch/fitness tracker	Signal-processing + online detection algorithm; anomaly detection	Pre-symptomatic physiological signature of infection (COVID-19)	N/A	Human HR/activity dynamic range	Detects physiological deviation but not pathogen-specific	63% pre-symptomatic detection in the cohort; 81% had alterations	Non-invasive, real-time, widely deployed, population scale	Not pathogen-specific; confounded by exercise/stress; device heterogeneity	[47]
Smartwatch + smartphone app (DETECT)	Multivariate classifier combining sensor metrics + symptoms (ML/statistical)	Distinguish symptomatic COVID+ vs. symptomatic COVID−	N/A	N/A	Improved discrimination when fusing sensor + symptom modalities	AUC = 0.80 (sensor+symptoms) vs. 0.71 (symptoms only)	Large cohort (30,529); scalable app-based collection	Self-report biases; device/platform heterogeneity	[50]
Biosensing wearable network (iPREDICT)—conceptual framework	Anomaly detection; Graph Neural Networks (GNNs); spatiotemporal models; federated learning (proposed)	Early outbreak/pandemic risk indicators	Conceptual; depends on underlying sensors	Population/spatiotemporal scale	Aims to improve selectivity via cross-user correlation and contextual data	Framework-level; specificity depends on model thresholds and context	Proactive outbreak detection: leverages crowd-sensed data and graph models	Requires standardized data, privacy-preserving infra, and broad adoption	[69]
Wearable biosensors + ICT (systematic review)	Surveyed ML/DL across studies (SVM, RF, CNN, ensemble, anomaly detection)	Patient deterioration, infection monitoring, remote triage	Varies by device/study; review-level	Device-dependent	Improved with multimodal fusion	Varied across included studies; heterogeneous reporting	Comprehensive mapping of ICT + wearable solutions; identifies effective strategies	Heterogeneous methods and metrics; variable evidence quality	[71]
IoT-based smart health monitoring system (prototype)	Rule-based alerts; proposed ML integration	Physiological indicators of infection/severity	Reported relative errors vs. commercial devices: HR 2.89%, Temp 3.03%, SpO_2_ 1.05%	Clinical vital sign ranges	Good for gross physiological changes; limited pathogen specificity	Not explicitly quantified	Low-cost, suitable for rural deployment; cloud storage for longitudinal tracking	Requires connectivity; privacy/security and calibration concerns	[74]
Smartwatch + explainable unsupervised learning pipeline	Unsupervised clustering; validated with supervised classifiers; GPT-3 for interpretation	Physiological anomaly clusters indicating infection (COVID-19)	N/A (clustering/classification task)	N/A	Clusters capture anomalies but need clinical labels for disease specificity	Supervised validation: accuracy 0.884 ± 0.005; precision 0.80 ± 0.112; recall 0.817 ± 0.037	Reduces reliance on labeled data; explainability aids clinician trust	LLM interpretation may introduce noise; cohort-dependent	[75]
24 h Holter ECG (clinical-grade HRV monitoring)	Statistical analysis (no ML reported)	Autonomic dysregulation in Post-COVID-19 Syndrome (PCS)	N/A	24 h monitoring window	HRV is sensitive to autonomic changes but not disease-specific	PCS patients showed significant HRV alterations vs. controls (after correction)	An objective clinical biomarker for autonomic dysfunction	Requires clinical equipment and interpretation; not a consumer wearable	[76]
Non-contact infrared thermometers (NCIT)—screening	ROC analysis and thresholding (no AI)	Fever detection as a proxy for infection	NCIT resolution ~0.1 °C	Skin temperatures ~30–40 °C	The neck site had the highest accuracy among the sites tested	Triple neck detection sensitivity up to 0.998; accuracy reduced at ambient < 18 °C	Fast, contactless, scalable for mass screening	Many infections are afebrile; ambient and site dependence; not pathogen-specific	[84]
IoT + Cloud + AI framework review for self-monitoring (5G enabled)	Survey of ML/AI approaches, cloud/edge analytics architectures	Physiological indicators associated with COVID-19/self-diagnosis	Device-dependent (review)	Varies with sensors	Multi-modal fusion is proposed to increase selectivity	Not experimentally quantified (review)	Comprehensive technology stack view; emphasizes low-cost cloud analytics and 5G benefits	Privacy, data security, deployment, and standardization challenges	[85]
UAV-mounted thermal camera (aerial thermal imaging)	Computer vision/deep learning classifiers for face detection, mask detection, temperature anomaly detection (hybrid ML/DL)	Fever screening/identify potentially febrile individuals in crowds (COVID-19 triage)	Thermal camera resolution dependent; not reported as concentration LOD (temperature resolution typical ~0.1 °C)	Up to drone operational range; system table: drone payload 2 kg, flight time 30–35 min (per paper)	Detects elevated skin temperature; not pathogen-specific; can include false positives (environmental effects)	Overall average accuracy reported ~99.5% for the proposed pipeline in test scenarios (paper reports high accuracy for detection tasks)	Rapid, contactless mass screening; can cover large crowds; includes mask detection	Skin temp not always reflective of core temp; environmental/ambient effects; cannot confirm infection	[90]
Smartphone onboard sensors (conceptual/app)	Deep learning frameworks (CNN/RNN/hybrid DL) for classification; proposed DL pipeline	Preliminary diagnosis/screening for COVID-19	N/A (classification task); performance metric: reported overall accuracy ~79% using smartphone sensors	User-device range (onboard sensors)	Depending on features used; cough/audio may be confounded with other respiratory illnesses	Not universally reported; overall accuracy 79% reported in this study	Widely available, low-cost, quick-deployable screening without medical tests	Requires labeled data, user compliance, false positives/negatives, and variability across devices	[93]
Contact piezoelectric sensor (and ultrasonic non-contact) for respiration	Logistic regression for classification of respiratory disease from collected vitals (simple ML)	Respiratory rate monitoring and screening for respiratory disease	N/A (physiological metric); device accuracy reported: overall device accuracy 96.58% for RR measurement	Respiratory rates in the typical human range (~5–40 bpm)—device validated on patients	Detects abnormal respiratory rate patterns; not disease-specific	Logistic regression classifier achieved 88% accuracy (5-fold CV) for respiratory disease detection	Low cost, accurate RR measurement, suitable for continuous monitoring	Contact sensor required; placement sensitivity (best positions vary by BMI); may be uncomfortable for long-term use	[96]
FMCW radar (non-contact)	Stacked ensemble ML models (ensemble of MLR, DT, RF, SVM, XGB, LGBM, CatBoost, MLP) and proposed Neural Stacked Ensemble Model (NSEM)	Classify respiratory behavior/detect abnormal breathing patterns (COVID-19 supervision)	Not concentration-based; radar sensitivity to chest micro-displacement; not reported as LOD	Room-scale; can detect multiple subjects and AoA separation	Can separate multiple objects and breathing characteristics; robust to lighting/privacy issues compared to the camera	The best model (NSEM) achieved 97.1% accuracy in experiments	Non-contact, privacy-preserving, can monitor multiple subjects simultaneously, with high accuracy reported	Requires RF hardware, signal interference, and may need careful calibration and line-of-sight	[99]
Wearable biosignal sensors (ECG, PPG, sEMG) for RR prediction	Deep learning: LSTM, Bi-LSTM, Attention LSTM, CNN-LSTM, ConvLSTM; Bi-LSTM with Bahdanau attention best	Accurate respiratory rate prediction from biosignals	N/A (physiological regression task); MAE reported as performance metric (best MAE 0.24 ± 0.03 for PPG + ECG dataset)	Depends on dataset; models evaluated on clinical/public datasets	Model differentiates RR patterns effectively; sensor-dependent noise affects selectivity	Performance reported as MAE; no binary specificity since regression task	High accuracy RR prediction with deep models; works across ECG/PPG/sEMG data	Require quality biosignals; model complexity and computational needs; window length affects performance	[100]
Imaging (chest X-ray) based diagnostic tool (hospital imaging)	Deep learning: transfer learning with CNNs (InceptionV3, ResNet50, Xception) and Vision Transformer (ViT)	Automatic COVID-19 detection and classification from CXR images	N/A (imaging classification)	Image-level classification, dependent on dataset quality and radiographic features	ViT showed superior ability to distinguish four classes vs. CNNs	Vision Transformer achieved a test accuracy of 99.3% (reported), outperforming ResNet50 (85.58%) in their experiments	High diagnostic accuracy reported (on their dataset); rapid automated triage potential	Requires clinical imaging equipment; dataset biases and limited generalizability; high accuracy may not generalize to diverse populations	[101]
Various wearables + smartphone/camera-based approaches (review)	Survey of ML/DL methods: CNN, RNN, image/signal processing, anomaly detection, explainable AI	Remote monitoring of vital signs for COVID-19 screening and monitoring	Varies by modality; review summarizes methods rather than specific LODs	Device-dependent; many methods are suitable for smartphone deployment	Varies; methods may struggle to be disease-specific, but useful for anomaly detection	Varied across studies; review discusses strengths and limitations (no single specificity value)	Enables remote, low-cost monitoring using ubiquitous devices; discusses practical deployment challenges	Heterogeneous literature; privacy and data-quality concerns; not yet clinical-grade across the board	[102]
Wearable IoT sensors for remote patient activity monitoring (multi-sensor wearables)	Proposed CNN-UUGRU deep model (convolution + updated gated recurrent units) for activity recognition	Activity recognition, remote patient monitoring, and detection of confinement breaches for quarantined patients	N/A (activity recognition/vital signs)	Wearable/device dependent; remote cloud connectivity via IoT	High for activity classes (model accuracy reported)	Reported performance: accuracy 97.7%, precision 96.8%, F-measure 97.75% on evaluated datasets	Integrated IoT-stack with high activity classification accuracy; cloud alerts and GPS tracking enable quarantine monitoring	Privacy concerns, connectivity needs, sensor calibration, and battery constraints	[103]

## 4. Artificial Intelligence-Based Wearable Sensing Technology for Diabetes Management

**Figure 10:**
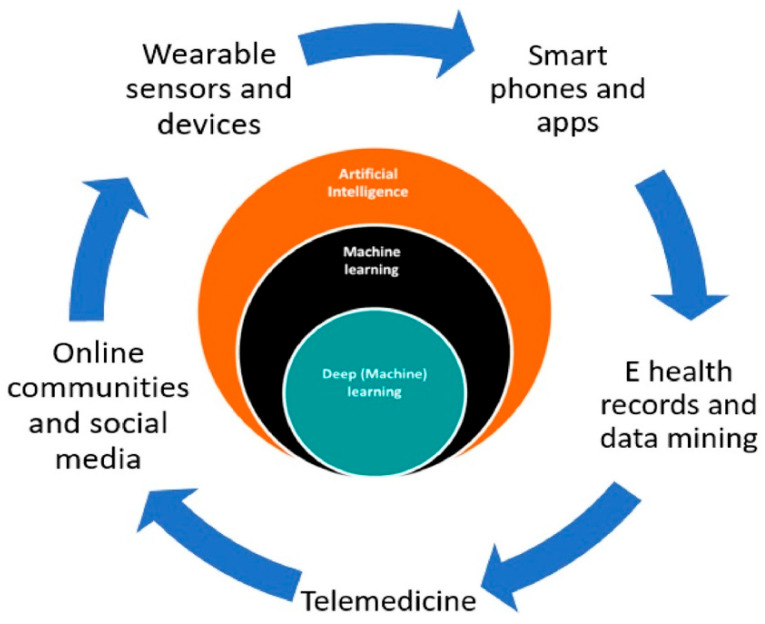
Use of AI in diabetes care. Reproduced with permission from ref. [115]. Copyright 2020 Elsevier.

Diabetes, which affects millions of people worldwide, is a chronic metabolic disease marked by the body’s failure to control blood glucose levels. This can be attributed to either insulin resistance (Type 2) or insufficient insulin production (Type 1) [111]. As a vital tool for managing diabetes, Continuous Glucose Monitoring (CGM) systems designed with the help of wearable sensing technology allow real-time surveillance of blood sugar levels with little need for manual intervention [112]. Based on electrochemical, optical, microneedle, and innovative sweat-based sensing technologies, etc., these devices offer a more sophisticated and practical substitute for conventional finger-stick glucose monitoring methods [113]. Their accuracy and user comfort have increased drastically, providing a more comprehensive approach to diabetes management. Wearable technology also progressively incorporates sensors for other physiological information, such as heart rate and physical movement [114]. Integrating Artificial Intelligence (AI) with these wearable devices to perform complex data evaluation and forecasting models has revolutionized diabetes management (Figure 10) [115]. AI algorithms, such as ML and DL, process large volumes of real-time data from wearables to forecast glucose trends, identify abnormalities, and propose personalized insulin dosage [116]. Closed-loop insulin administration systems have also benefited from the deployment of AI-driven models, which automate insulin adjustments and enhance glycemic control with minimal human intervention. This review highlights recent progress in wearable sensing technologies using artificial intelligence (AI) for managing diabetes, emphasizing sensing technology in subsequent subsections [117].

### 4.1. Continuous Glucose Monitoring (CGM) Devices

#### 4.1.1. Electrochemical Sensors

Electrochemical sensors are crucial for continuous glucose monitoring (CGM) systems due to their high sensitivity and real-time glucose detection. These sensors measure glucose oxidation in interstitial fluid and produce electrical signals proportional to the amount of glucose present [112]. The simplicity and comfort of continuous monitoring for diabetes patients have improved over time as these sensors have become smaller and integrated into wearable devices [113]. However, the sheer amount of data these sensors generate makes real-time analysis and interpretation more difficult. To address this, Artificial Intelligence (AI)-driven control algorithms, such as machine learning (ML) and reinforcement learning models, have been integrated with electrochemical sensors to process data and predict glucose trends. These AI models provide sophisticated insights into glucose changes that may not be evident in conventional monitoring systems, assisting in the early diagnosis of hypo- and hyperglycemic episodes and helping track insulin changes [118]. AI’s ability to estimate glucose levels up to 60 min in advance has dramatically improved the accuracy and therapeutic utility of these systems, giving diabetes patients more effective and individualized management options [119]. This integration enables automated, data-driven decision-making for more efficient glucose management, representing a significant advancement in diabetes treatment.

There is a challenge in determining which AI technique best mitigates the confounding effects of temperature drift in wearable electrochemical glucose sensors [120]. Calibration models using ML, such as support vector regression and random forests, are widely used to model the nonlinear relationship between sensor output and temperature variation. The second approach is a deep learning method, such as convolutional neural networks and recurrent neural networks, which can understand the temporal patterns of glucose signals in conjunction with temperature fluctuations, thereby enhancing robustness in dynamic real-world environments. This is a sensor fusion framework that integrates AI techniques to combine multisensory data, helping to disentangle the effect of temperature using multimodal neural networks. Adaptive algorithms, such as online learning and adaptive filtering, enable real-time correction of temperature drift by continuously updating the model with new data from the individual wearer. Among all, Deep learning, especially CNN-LSTM hybrid models with sensor fusion, has great potential in recent studies to mitigate the temperature drift confounding effect while maintaining real-time performance [121].

#### 4.1.2. Optical Sensors

Optical sensors can measure glucose levels by detecting how light is scattered or absorbed by biological tissues. This capability has made them a viable and prominent non-invasive tool for glucose monitoring. Techniques like fluorescence, Raman spectroscopy, and near-infrared (NIR) spectroscopy serve as the building blocks of optical sensors. These techniques can minimize the discomfort associated with invasive methods like standard finger-prick testing [122]. These sensors can provide continuous monitoring by evaluating variations in the optical characteristics of skin or interstitial fluids and correlating them with glucose concentrations. However, the primary concern in using optical sensors is reducing interference from other physiological factors, such as temperature, tissue heterogeneity, and motion artifacts [123].

Here, artificial intelligence (AI) models can also be incorporated to handle the complex data generated by optical sensors and improve their performance. Artificial intelligence models aid in noise reduction and interference compensation, providing more precise glucose measurements. For instance, light sources spanning 18 wavelengths from 410 to 940 nm can improve glucose detection sensitivity and selectivity in aqueous solutions. Errors arising from variations in blood and tissue components across individuals can be mitigated by using multiple wavelength measurements. Five ML techniques are investigated for glucose prediction: regression analysis, k-nearest neighbors, decision trees, and neural networks [124]. Pal et al. proposed a blood glucose sensing model that uses a digital camera, a magnetic field, and records speckle patterns from a finger using a laser [125]. ML and DNN techniques are used to preprocess and evaluate recorded video patterns to identify glucose levels. AI models trained on optical sensor data have been used to accurately estimate glucose levels, even in the presence of tissue scattering and skin pigmentation [126]. AI has shown promise in real-time glucose monitoring and in minimizing the need for invasive procedures when combined with wearable optical sensors. This connection enables more accurate, continuous, and user-friendly glucose management, a significant advancement in the field.

#### 4.1.3. Microneedle Sensors

**Figure 11:**
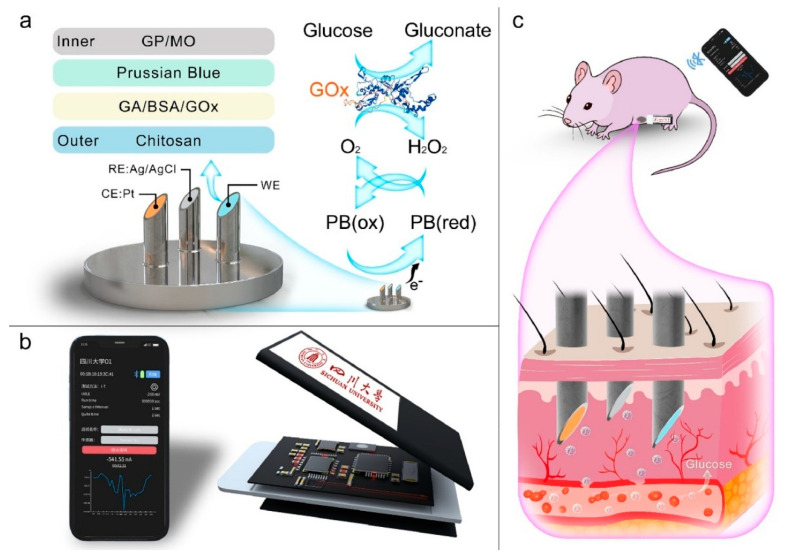
(**a**) Wearable microneedle-based sensing system for CGM, (**b**) illustration of profile structure and program control interface, and (**c**) concept of wearable CGM system on a Sprague Dawley rat. Reproduced with permission from ref. [128]. Copyright 2023 Elsevier.

Microneedle sensors can access interstitial fluid just beneath the skin’s surface without causing discomfort, and their minimally invasive nature has made them a popular choice for continuous glucose monitoring (CGM). Compared to conventional finger-prick methods, these sensors use microscopic needles to puncture the skin, offering constant and real-time glucose monitoring with greater precision [127]. Microneedles are perfect for wearable applications in managing diabetes since they are frequently combined with electrochemical sensing technologies to measure glucose concentrations in interstitial fluid. Li et al. reported a microneedle-based CGM platform developed using a three-electrode electrochemical biosensor and evaluated the sensing efficacy (Figure 11). Incorporating PB, Gox, and chitosan enables the formation of a stable 3D network that exhibits a sensing range of 0.25 to 35 mM, high sensitivity, and anti-interference properties. They have performed in vivo analysis using the diabetes rate to evaluate therapeutic efficacy. More interestingly, it provided accurate real-time glucose, which is further consistent with commercial meters [128]. A biomimetic microneedle theragnostic platform (MNTP) uses microneedle arrays for simultaneous glucose and ion monitoring and on-demand skin penetration to provide accurate diabetes treatment [129]. Utilizing hybrid carbon nanomaterials to functionalize the epidermal sensor allowed for the detection of oxygen-rich interstitial fluid. In an innovative approach to manufacturing microneedle sensors, 3D printing, microfabrication, electrodeposition, and enzyme immobilization were employed to create a PMMA-based microneedle detector for glucose. In vitro evaluation revealed that the sensor had an excellent selectivity and repeatability, a linear range of 1.5 to 14 mM, a sensitivity of 1.51 µA mM^−1^, and a detection limit of 0.35 mM [130].

Microneedle CGM patches can maintain sterility for a week of wear because they are generally fabricated under aseptic conditions and packaged in sterile, single-use formats, thereby reducing the risk of contamination. Biocompatible polymers, hydrogels, and coatings infused with antibacterial agents further reduced microbial colonization during prolonged skin contact. The interface between the microneedle and the skin forms shallow, self-sealing microchannels after removal, reducing the risk of infection. Further CGM patches are integrated with protective adhesive films, which act as a barrier against dust, sweat, and external microbial entry [131]. Week-long sterility and safety have been validated in several pilot and clinical studies, with low irritation and infection rates observed. Hence, CGM patches are ideal for long-term applications.

Artificial Intelligence (AI) has improved data processing and predictive accuracy, greatly expanding the potential of microneedle sensors. He et al. discussed how analyzing the continuous data produced by microneedle sensors enables machine learning (ML) models, such as support vector machines and neural networks, to predict glucose trends and aid in the more efficient management of glucose fluctuations [132]. To maximize fluid collection, the physical parameters of microneedle (MN) designs were optimized by integrating machine learning (ML) models with finite element methods (FEMs) [133]. ML algorithms were trained on FEM simulations with varying geometric parameters, and the best predictions were obtained with decision tree regression (DTR). Machine learning techniques can optimize MN designs for targeted drug delivery and point-of-care diagnostics in wearable devices. The accuracy of glucose readings can be further improved by using AI-based algorithms that can adjust for changes in the sensor environment, such as skin condition and body movement [134]. Diabetes-related wounds are difficult to heal because of inflammation and poor tissue regeneration. Xue et al. discovered that a wound-healing drug, Trichostatin A (TSA), targets histone deacetylase 4 (HDAC4) for diabetes treatment using AI-assisted bioinformatics [135]. A novel, minimally invasive therapeutic strategy was developed using a microneedle (MN)-mediated patch loaded with TSA, which reduced inflammation, improved tissue regeneration, and suppressed HDAC4.

### 4.2. AI-Driven Insulin Pumps and Closed-Loop Systems

Artificial intelligence (AI)-driven insulin pumps and closed-loop devices, also known as artificial pancreas, have become groundbreaking tools for improving diabetes care. These systems use intelligent algorithms, such as machine learning (ML) and deep learning, to autonomously adjust insulin delivery via insulin pumps based on real-time data from continuous glucose monitoring (CGM). Advanced control algorithms, including fuzzy logic, model predictive control (MPC), and proportional-integral-derivative (PID) controllers, provide the foundation of these systems’ technological architecture to balance current insulin dosage with historical trends [136]. Zhou and Isaacs stated that, based on the patient’s physiological feedback, these algorithms aim to keep blood sugar levels between 70 and 180 mg/dL, avoiding hypoglycemia and hyperglycemia [137]. According to the study conducted by Guzman Gomez et al., Artificial Intelligence techniques can further improve these control strategies, providing insulin more precisely and promptly while reducing the risk of hypo- and hyperglycemia [138].

Maintaining a balance between safety and efficiency is a significant challenge in AI-driven insulin delivery. To provide a solution for this, Zarkogianni et al. designed and evaluated a personalized insulin infusion advisory system (IIAS) that uses insulin pumps and continuous glucose monitors to provide real-time insulin rate estimations for patients with Type 1 diabetes [139]. Recurrent neural network and compartmental models are utilized along with a tailored glucose-insulin metabolism model and a nonlinear model-predictive controller (NMPC). The predictions were made based on patient data, including food consumption, glucose readings, and insulin infusion rates. Similarly, Ahmed et al. conducted a comprehensive study to ensure that AI systems must deliver the right amount of insulin to the patient, considering his/her food and metabolic activities like stress, physical activity, food consumption, etc. [140]. Basal insulin and high-concentration pre-meal injections are insufficient for adequate blood glucose management in diabetes because they may not provide long-term stability. Adaptive learning techniques also allow the system to learn from prior glucose trends, improving predicted accuracy and lowering the frequency of sensor recalibration [141]. A system that combines a pumping module, glucose sensor, and decision unit has recently been designed [142]. The decision unit utilizes artificial intelligence (AI) to analyze glucose data and determine the optimal insulin dosage. This method prolongs the time that blood glucose levels stay within a healthy range and improves insulin delivery. Researchers have investigated using Reinforcement Learning (RL) supported by the Markov Decision Process (MDP) model to modify insulin administration tactics in response to real-time glucose level feedback [143].

Although rule-based algorithms were the mainstay of early iterations of these systems, more recent developments have brought hybrid AI-driven models that integrate data-driven and control-theoretic methodologies. Boughton and Hovorka studied how these hybrid models better control blood glucose levels over time [144]. Additionally, AI-powered devices are increasingly customized, personalizing insulin administration protocols according to each user’s unique physiological requirements and lifestyle choices [145]. Nimri et al. conducted a non-inferiority test on an extensive dataset of 108 participants over six months to compare the insulin prescription levels of medical practitioners using traditional clinical methods with those prescribed by an automatic AI system [146]. The results reveal that predictions from AI-based glucose monitoring and insulin prediction systems are as accurate as those from medical experts, while being faster and more economical.

### 4.3. Non-Invasive Glucose Monitoring Wearables

#### 4.3.1. Smart Contact Lenses

**Figure 12:**
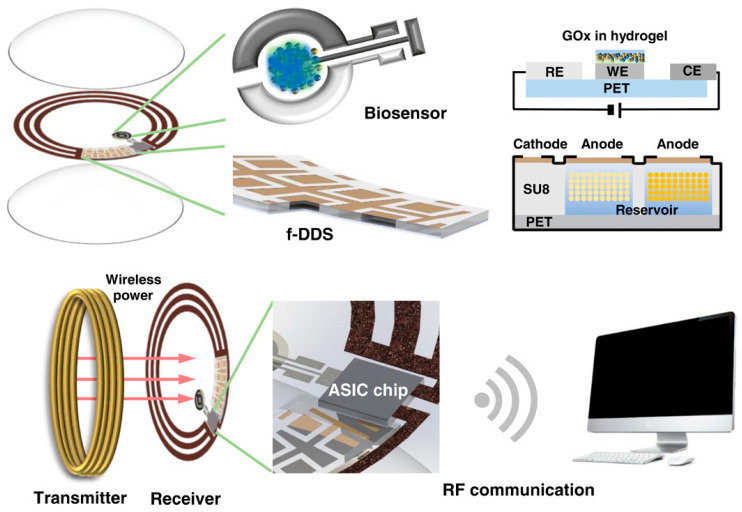
Smart contact lens for diabetes identification and treatment. Reproduced with permission from ref. [147]. Copyright 2020 American Association for the Advancement of Science Open Access.

**Figure 13:**
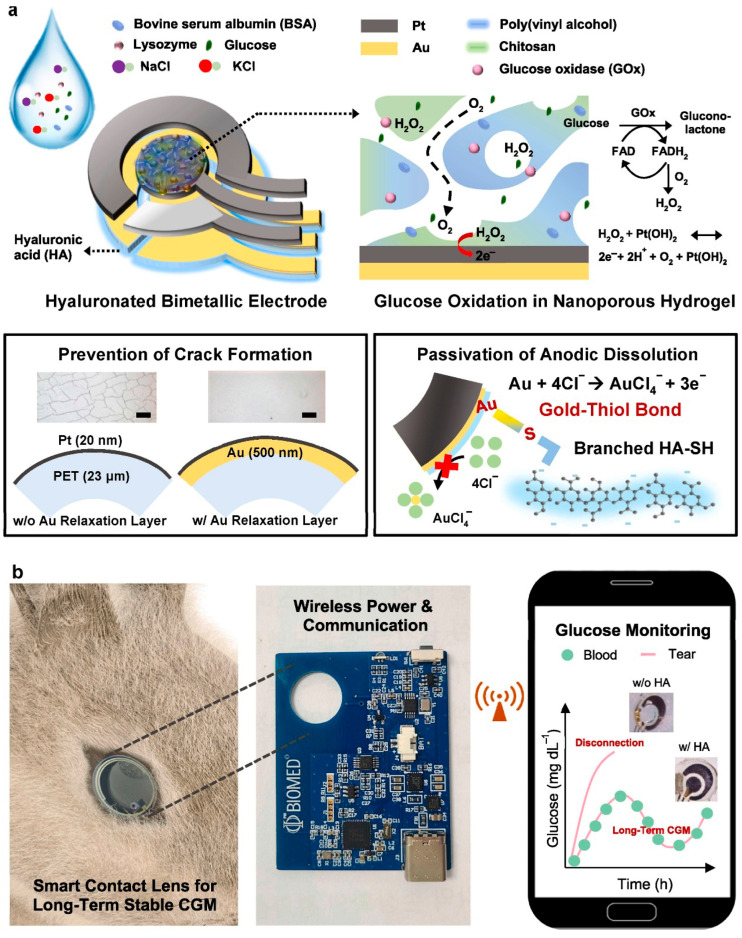
(**a**) Hyaluronated Au@Pt bimetallic electrodes schematic and working mechanism in smart contact lens, and (**b**) In vivo real-time glucose supervision. Reproduced with permission from ref. [151]. Copyright 2023 Elsevier.

In diabetes management, smart contact lenses are gaining popularity as a cutting-edge method for continuous and non-invasive glucose monitoring. Intelligent contact lenses can be an alternative to conventional blood glucose monitoring techniques; these glasses incorporate biosensors that detect glucose levels in tear fluid [147]. Keum et al. reported a wireless smart contact lens for non-invasive diagnosis and therapy of diabetes. The smart contact lenses comprise a biosensor for real-time glucose detection, a flexible drug-delivery system, an ASIC microcontroller chip for signal processing, and a radio-frequency communication module for transmitting data to external devices. All these components are shown in Figure 12. They designed biosensors by immobilizing glucose oxidase on a chitosan-PVA hydrogel, which can detect tear glucose levels of 5–50 mg/dL, with amperometric current changes ranging from 0.41 to 3.12 μA. It showed a strong correlation with tear and blood glucose levels in diabetic rabbits. F-DDS enables the release of genistein and metformin via voltage-triggered dissolution of a gold membrane. They achieved 90% of the therapeutic release. Further, this device is stable for over 63 days [147]. This device combines biosensing, microelectronics, and wireless communication, and is based on an AI-assisted theragnostic device. However, using opaque, fragile components to enable an electronic device to function poses a problem for a bright contact lens, which might cause eye injury and block vision. Furthermore, user external activities are also hindered by the large, heavy equipment used to measure sensor signals. Park et al. proposed a soft, fabric-based intelligent contact lens that combines transparent, flexible nanostructures for wireless power transmission, real-time signal display, and glucose monitoring without impairing vision, thereby addressing this issue [148]. To improve smart contact lenses, ultra-thin, flexible circuits, and wireless transmission systems deliver better results, powering these lenses to enable real-time data transmission to mobile devices for continuous monitoring [149]. A FIP chip with blue Mini-LEDs (FC blue Mini-LEDs MOI) has been fabricated for glucose detection. That included a tiny spectrometer, collimating lenses, an optical fiber, and a 459 nm center-wavelength Mini-LED [150]. Utilizing optical technology, this system enables precise glucose monitoring. An Au@Pt bimetallic electrode modified with hyaluronate (HA) for accurate, long-term, continuous glucose monitoring (CGM) has been incorporated into smart contact lenses [151]. The bimetallic structure improves hydrogen peroxide breakdown and charge transfer for efficient glucose detection, while the HA coating inhibits chloride from dissolving the Au electrode. Smart contact lenses not only enable continuous glucose monitoring but also aid in the treatment of diabetic retinopathy (Figure 13). Theo’s improved diabetes management enables lower risk of hypo- or hyperglycemia; AI algorithms are being integrated into these systems to forecast glucose fluctuations and offer personalized suggestions [152].

#### 4.3.2. Sweat-Based Sensors

**Figure 14:**
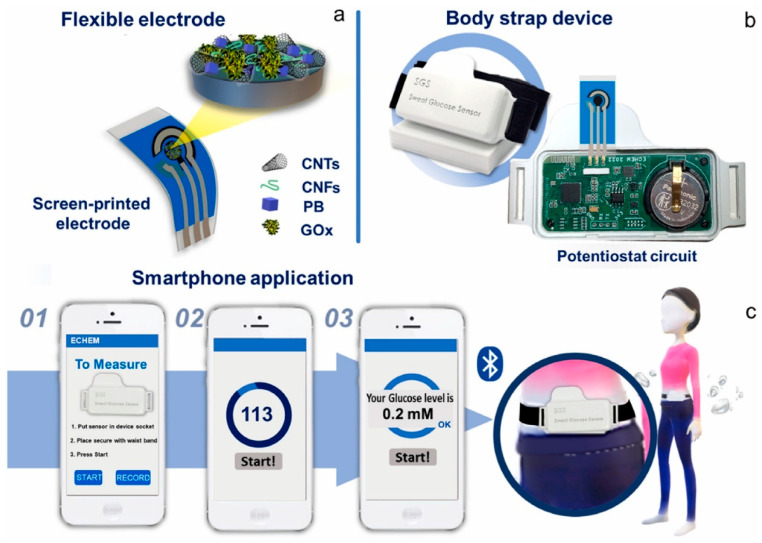
(**a**) Wearable body strap for glucose sensing, (**b**) illustration of body strap, and (**c**) smartphone program by Bluetooth. Reproduced with permission from ref. [158]. Copyright 2024 Elsevier.

Sweat-based sensors have become a viable non-invasive alternative to blood-based techniques for continuous glucose monitoring in diabetes care. These sensors measure the composition of human sweat, which contains glucose at concentrations that correlate with blood glucose levels [153]. However, the lower glucose concentration in sweat compared to blood poses a significant technical hurdle, necessitating sensitive detection methods [154]. To address this, Kim et al. attempted to incorporate sweat sensors into flexible, wearable substrates, such as hydrogels or stretchable electronics, along with enzymatic glucose detection methods, such as glucose oxidase, to ensure comfort during prolonged use. According to Sankhala et al., integrating Artificial Intelligence with these sweat-based glucose monitoring sensors has become a significant breakthrough in overcoming the technical difficulties caused by sweat’s lower glucose concentration, which results in frequent fluctuations when evaluating the metrics [149]. AI can improve the sensor’s predictive power by fine-tuning the relationship between blood glucose and sweat glucose levels using analysis of large datasets, as described [155]. Using machine learning models, such as neural networks, deep learning, and dynamic pattern recognition, to analyze continuously streaming data has become possible, enabling predictive analytics of glucose [156]. They have developed a body strap device for glucose sensing, integrated with smartphone applications via Bluetooth. The device comprises flexible electrodes as sensing strips, a potentiostat circuit within the device for electrochemical detection, and a mobile phone with an application for signal processing and readout. The body strap sensing device is illustrated in Figure 14. The body strap sensing device detects glucose in the 0.1 to 1.5 mM range with a cut-off of 0.3 mM, enabling it to distinguish between diabetes and non-diabetes. The ML model, XGBoost regression, was used to correlate the electrochemical response and improve accuracy. Altogether, offer non-invasive, real-time glucose monitoring via a smartphone application with high precision and stability for up to 21 days [156]. An explainable deep learning (DL) based programmable colorimetric device is created to precisely classify and quantify sweat sample data [157]. In conjunction with a convolutional neural network (CNN), the chip accurately identified and quantified glucose, pH, and lactate from 4600 recorded colorimetric response photos using sodium alginate gel capsules as indicators. In this challenge, CNN fared better than other DL and machine learning methods. By learning from user data, AI algorithms can adapt to variables that affect glucose readings, like perspiration rate, hydration levels, and physical activity [158]. Moreover, AI can help with real-time alarms by identifying patterns in blood sugar levels and predicting hypo- or hyperglycemic episodes before they occur.

It is interesting to note false positive/negative biomarker detection, such as sweat glucose and blood glucose. Several factors influence it, including physiological variability, environmental and user factors, algorithmic mitigation, and validation approaches. Sweat and interstitial fluids may not have a linear correlation with blood glucose. This may lead to discrepancies in the detection and interpretation of accurate glucose levels. Temperature, hydration, skin pH, and motion artifacts can contribute to noise and errors in wearable sensing output. Hence, there is a risk of false-positive or false-negative data. AI algorithms, such as sensor fusion, error-correction models, and machine learning calibration methods, may help reduce false positives and negatives, thereby enhancing data reliability. Cross-verification against a gold-standard clinical measurement, such as blood-based assays, further enhances reliability before clinical application.

#### 4.3.3. Multi-Parameter Wearables: Smartwatches and Fitness Trackers

**Figure 15:**
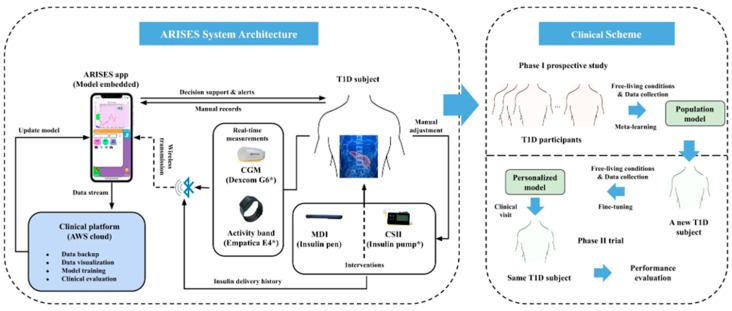
Illustration of the ARISES system and clinical scheme. Reproduced with permission from ref. [165]. Copyright 2022 Springer Nature Open Access.

In today’s world, smartwatches and fitness trackers are essential for managing diabetes since they can continuously monitor multiple physiological parameters and provide immediate feedback. These wearables have sensors that can noninvasively monitor blood glucose levels, physical activity, heart rate, and sleep patterns to help users better understand their overall health and manage their diabetes [159]. For example, wearable technology like the Apple Watch and Fitbit can track blood sugar levels using sweat-based sensors or third-party apps connected with continuous glucose monitors (CGMs) [160]. Artificial intelligence (AI) is the critical component of harnessing the potential of these wearables; according to Makroum et al., using AI models, users can make rational decisions on insulin dosage, exercise, and dietary modifications by predicting glucose changes based on an individual’s activity levels, meal intake, and previous data [161]. Therefore, AI has also transformed these wearable devices into THINKables [162]. Using personal health devices like smartphones and smartwatches, Ramesh et al. suggest an end-to-end remote monitoring paradigm for diabetes risk identification and supervision [163]. After pre-processing the data, a support vector machine model trained on the Pima Indian Diabetes Database is used to forecast the risk of diabetes. The framework ensures unobtrusive monitoring, cost-effectiveness, and device interoperability while facilitating informed medical decisions and slowing the progression of diabetes. Another significant development in diabetes management is the integration of Fitbit trackers and smartwatches with smartphone apps and cloud platforms that store and process data. AI-powered systems leverage this data to produce predictive analytics and customized health reports, giving consumers valuable insights [164]. People with type 1 diabetes (T1D) must monitor their blood sugar levels on a lifelong basis to prevent harmful glycemic issues. To help with this, Zhu et al. designed ARISES (Figure 15) (Adaptive, Real-time, and Intelligent System to Enhance Self-care), a DL algorithm-powered smartphone platform that predicts glucose levels and identifies hypo- and hyperglycemia using information from meal and insulin entries, wristband sensors, and continuous glucose monitors (CGM) [165]. Various AI-based wearable sensing devices for Diabetes management and their features, such as type of wearable devices, kind of AI, target analyte, limit of detection, detection range, selectivity, specificity, pros and cons, are summarized in Table 2.

**Table 2:** Summary of literature related to various AI-based wearable sensing technologies for diabetes management.

Type of Wearable Sensor	AI/Algorithm Used	Target Analyte	Limit of Detection	Detection Range	Selectivity	Specificity	Pros	Cons	Ref.
Noncontact speckle-based optical finger sensor	Machine Learning classifiers; tested DNNs (ML outperformed DNNs)	Plasma glucose levels (classification)	Not reported as LOD; classification accuracy reported	Physiological glucose ranges (capable of classifying hypo/standard/hyper bands)	Improved by magneto-optic modulation + preprocessing	High classification accuracy reported (ML > DNN in this dataset)	Totally noncontact; low-cost hardware; AI improves selectivity and sensitivity	Needs larger, diverse cohorts; environmental and motion sensitivity	[125]
Fully integrated microneedle continuous glucose monitor (MN-CGM)	Signal processing + potential ML for calibration discussed (not primary focus)	Glucose in ISF (clinical CGM range)	LOD not explicitly stated; wide linear range demonstrated	0.25–35 mM (wide clinical range)	High via enzyme specificity and PB mediator	Good correlation with commercial glucose meters in animal studies	Wide linear range, stable, suitable for real-time continuous monitoring	Implantable/microneedle invasiveness (minimally), enzyme stability over long term	[128]
Swelling microneedle patch delivering TSA for wound healing (AI-guided)	AI-assisted bioinformatics, molecular docking, and sequencing analysis	HDAC4 and associated inflammatory pathways	N/A (therapeutic focus)	N/A	High target specificity per bioinformatics/docking	Validated in vitro and in vivo models	AI-guided drug repurposing; minimally invasive targeted therapy	Not a continuous wearable sensor; translational work needed	[135]
Survey/review of BG prediction methods (not a single device)	Surveyed ML/DL methods: SVM, RF, ANN, LSTM, hybrid models	Predicted blood glucose levels and adverse events	N/A (review)	Depends on underlying sensors (typical CGM ranges)	Varies by model and feature set	Discussed in literature; performance metrics summarized across studies	Comprehensive overview of trends, input features, modeling techniques, and challenges	Not original experimental device data; heterogeneity across studies	[140]
AI-based insulin dose optimization (AI-DSS) integrated with insulin pumps and CGM	Proprietary AI-DSS (DreaMed Advisor Pro)—rule-based + ML components	Glucose control and insulin dose settings	CGM device-dependent	CGM operational range (e.g., 40–400 mg/dL)	Effective for individualized insulin titration	Non-inferior to physician adjustments in RCT (safety endpoints)	Reduces clinician workload; safe and effective per multicenter RCT	Requires accurate CGM and adherence; regulatory and integration aspects	[146]
Smart, soft contact lens with integrated glucose sensor and display	Signal processing + ML-based filtering for improved readout	Glucose in tear fluid	Reported ~30 µM	Approximately 50–500 µM in tears	High via enzyme specificity	Correlating with blood glucose with time lag consideration	Fully integrated, transparent, wireless, real-time visualization	Tear-blood correlation lag; fabrication complexity; comfort and safety considerations	[148]
On-demand sweat glucose EIS sensor with ML reporting (SWEET platform)	Decision Tree Regression for prediction and mapping to glucose	Sweat glucose (mg/dL)	RMSE ~0.1 mg/dL in reported tests	Reported 1–4 mg/dL (physiological sweat glucose range)	High via affinity probe; reduced interferents	R^2^ = 0.94 in validation datasets	Completely non-invasive, frequent sampling (1–5 min), ML enables robust reporting	Inter-subject variability; dependency on sweat availability; needs broader clinical validation	[149]
Systematic review: ML and smart devices for diabetes management	Survey of ML techniques: SVM, RF, ANN, ensemble, DL	Glycemic events, BG prediction, complications detection	Not applicable	Dependent on the specific device/sensor	N/A (review)	Summarized per the study surveyed	Comprehensive synthesis of 89 studies; highlights trends and gaps	Heterogeneity in study designs, limited to 2011–2021	[161]
Remote healthcare monitoring framework using wearables for diabetes prediction	Support Vector Machine (SVM) classifier	Diabetes risk/classification	N/A (classification task)	Binary or risk-score outputs	Accuracy 83.2% (10-fold CV)	79% specificity; sensitivity 87.2%	Vendor interoperability; remote monitoring potential	Limited dataset generalization; relies on input quality	[163]
ARISES: Multi-modal wearable + DL platform for T1D self-management	Deep Learning model (RNN/LSTM-based) for 60 min prediction horizon	Glucose forecasting	RMSE = 35.28 ± 5.77 mg/dL (60 min horizon)	Reported clinically relevant ranges; the model reduces prediction error with wristband data	Improved detection of events (Matthew’s coefficients reported)	Matthew’s coefficients: 0.56 (hypo), 0.70 (hyper), indicating a reasonable specificity/sensitivity balance	Demonstrated reduction in prediction errors when including wearable data; implemented in smartphone app	Small study cohort (12 adults in longitudinal study for model development); needs larger validation	[165]

## 5. Artificial Intelligence-Based Wearable Sensing Technology for Cancer Management

Cancer is a serious global health concern, resulting in many deaths in the entire world. Early detection and continuous monitoring are crucial for enhancing patient treatment outcomes and survival. Wearable sensing devices have significantly transformed cancer management by enabling real-time tracking of physiological changes and biomarkers associated with cancer. Electrochemical sensors and intelligent textiles have been used to identify cancer indicators like CA-125 for ovarian cancer and exosomes in sweat [166]. However, skin cancer can be identified by examining tissue anomalies with the help of optical sensors [167]. Meanwhile, other wearable devices can track several vital indicators to monitor the course of the disease and its treatment. By reducing the frequency of hospital visits, these sensors offer a personalized, practical approach to cancer management. The potential of these wearables has been further increased by incorporating artificial intelligence (AI) [168]. Large amounts of data generated by these sensors are processed by AI systems, which identify patterns and improve the precision of diagnoses. AI has been applied in multimodal sensing systems, cancer treatment side effects monitoring, and cancer recurrence prediction [169]. AI-driven systems have effectively integrated multiple sensor outputs to anticipate treatment challenges and identify cancer biomarkers more precisely. Wearable technology and artificial intelligence (AI) provide a more comprehensive, data-driven approach to cancer care, enhancing early detection, individualized treatment, and overall patient outcomes.

### 5.1. Biosensing Technology-Based Wearables

**Figure 16:**
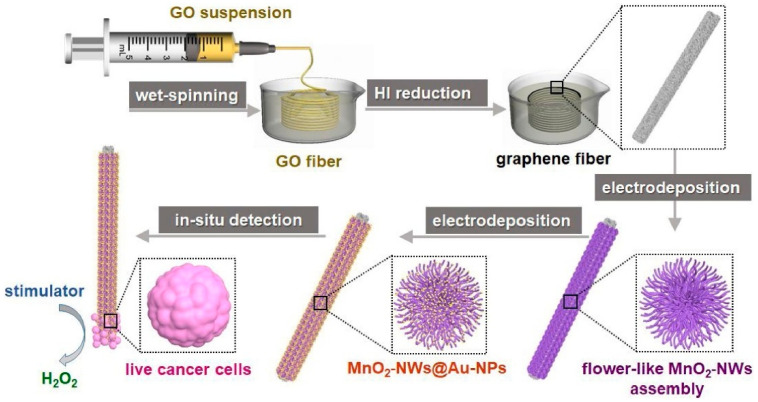
Synthesis method of MnO_2_-NWs@Au-NPs/GF microelectrode for live cells detection. Reproduced with permission from ref. [172]. Copyright 2020 Elsevier.

Wearable sensor-based biosensing technology is a revolutionary device that continuously and non-invasively monitors biological signals and biomarkers from the human body. These sensors can detect proteins, enzymes, and metabolites linked to cancer in bodily fluids, including sweat, saliva, and interstitial fluid [170]. They are often combined with modern material substrates and nanotechnology [171]. Their significance in cancer care lies in their ability to provide real-time monitoring of the disease’s course and facilitate early cancer detection, thereby enabling assessment of treatment efficacy. Electrochemical sensors are prominent biosensors that have attracted significant attention in cancer management because they convert biological reactions into detectable electrical signals. Numerous researchers have analyzed various aspects of these sensors, including construction materials, performance, size, detected biomarkers, and applications. For instance, Zhao et al. designed an innovative high-performance microelectrode based on a wet-spun graphene fiber (GF) functionalized with a dual nanoenzyme comprising ultrafine Au nanoparticles (Au-NPs) wrapped in MnO_2_ nanowires. Figure 16 presents the step-by-step procedure for fabricating the MnO_2_-NWs@Au-NPs/GF microelectrode. Initially, GF was obtained via wet spinning, and subsequent chemical reduction yielded conductive GF. Electrodeposition was used to deposit MnO_2_-NWs and Au-NPs onto the GF surface. This microelectrode is flexible and enables real-time H_2_O_2_ detection in live cancer cells. It showed a high sensitivity of 32.9 µA/cm^2^ mM and a LOD of 1.9 µM. it is ideal for wearable, implantable, and portable biosensing applications [172]. This microelectrode is used to electrochemically detect hydrogen peroxide (H_2_O_2_), a biomarker released by living cancer cells. The suggested sensor has excellent potential for use in cancer cell monitoring applications. Similarly, to detect H_2_O_2_ and improve sensitivity, gold nanoparticles encapsulated in N-doped carbonized silk fibroin (AuNPs@CSF) were used to create a wearable electrochemical sensor with enhanced sensitivity and endurance on a modified silk fibroin substrate [173]. For improved sensing technology, high-density silicon microneedle arrays coated with gold (Au–Si–MNA) electrochemical transducer biomarkers have been developed for the extraction platform. This helps selective immunocapture and measurement of epidermal growth factor receptor 2 (ErbB2), a key breast cancer biomarker [174].

Carbon-based electrochemical biosensors represent a novel approach to cancer monitoring, owing to their excellent sensitivity, selectivity, and rapid reaction times. A deep study conducted by Karimi et al. to develop carbon-based sensors with an emphasis on their composition, methods of action, and potential for identifying cancer biomarkers and evaluating therapeutic outcomes [175]. Further improvements were made in the flexibility of wearable sensors and in the simultaneous identification of tumor markers in complex samples. They utilize ultrathin graphdiyne (U-GDY) and facilitate electron transport, enzyme loading, and detection sensitivity. The sensor provides and processes real-time electrochemical data via a smartphone [176]. At the same time, the DNA nano manager scheme facilitates signal amplification, making it a viable method for early cancer diagnosis and potentially flexible wearable applications.

**Figure 17:**
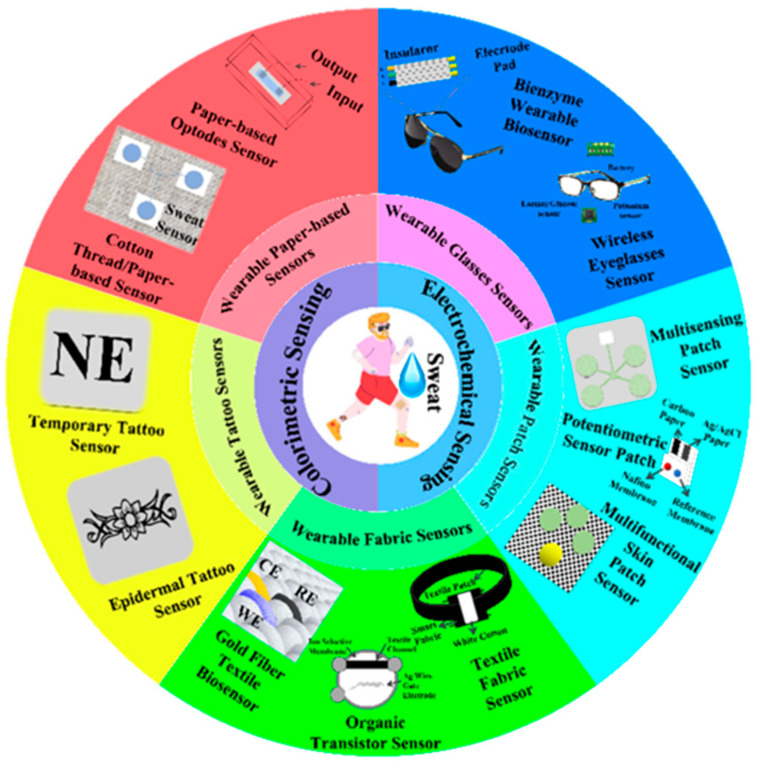
Sweat-based wearable electrochemical sensors, including wearable glasses, patches, fabrics, tattoos, and paper sensors. Reproduced with permission from ref. [177]. Copyright 2022 MDPI Open Access.

In addition to serving as a medium for interstitial fluid-like substances, human sweat also serves as an input to specific sensors that detect cancer biomarkers, including pH, lactate, and specific proteins linked to tumor activity. Sweat-based sensors utilize electrochemical and colorimetric sensing techniques to detect subtle changes in biomarker concentrations. As an illustration, Qiao et al. systematically reviewed the use of a sweat sensor and a flexible wearable platform to detect changes in lactate, a metabolic waste frequently high in cancer patients (Figure 17) [177]. The sensor was designed to achieve high sensitivity and specificity by incorporating functionalized nanomaterials. Existing wearable electrochemical systems rely on chemical or electrical stimulation, or high-intensity exercise to actively release sweat in low-perspiration environments, which poses challenges for continuous monitoring. Therefore, to deal with this issue, Saha et al. proposed a continuous sweat lactate monitoring technology that combines a wireless wearable potentiostat system for real-time tracking, a screen-printed electrochemical lactate sensor, and a paper microfluidic channel for osmotic sweat extraction [178]. The effectiveness of sweat management and biomarker detection is improved by this novel method.

In cancer management, incorporating AI with electrochemical sensors has proved to be an essential combat tool against the disease because of its ability to process and evaluate electrical signals [179]. This increases detection accuracy and reduces false positives, thereby improving the sensor’s efficiency. Artificial intelligence (AI) approaches, including regression, neural networks, association rule mining, and deep learning, can help identify intricate patterns in sensor data that are difficult to detect with traditional methods. For example, a novel biosensor is fabricated that uses machine learning (ML) methods in conjunction with green silver nanoparticles (GAgNPs) to detect canine mammary carcinoma biomarkers, such as MUC-1 in tissue samples and CA 15-3 in serum, with high sensitivity [180]. However, in urine cytology, low sensitivity poses significant difficulty in detecting urothelial cancer of the bladder (UCB). For that, a non-invasive technique has been introduced for bladder urothelial cancer (UCB) that uses machine learning (ML) in conjunction with micro-dimensional electrochemical impedance spectroscopy (µEIS) to differentiate between malignant and healthy urothelial cells [181]. Pneumatic valve-equipped flow cytometry systems increase sensitivity by maintaining tight, clog-free contact between cells and electrodes. Electrochemical sensors for identifying exosomes in bodily fluids have also been improved using AI-based signal processing. The characteristics and compositions of exosomes and their use in tumor diagnostics and treatment, especially in brain tumor cases, have been shown to distinguish between benign and malignant, allowing the detection of cancerogenic exosomes [182].

Medical researchers frequently use machine learning (ML) systems to analyze data collected by sweat-based sensors and to evaluate cortisol trends over time. A non-invasive sensor on a flexible, nano-porous substrate can passively detect cortisol in human sweat [183]. Sensor responses were recorded using Electrochemical Impedance Spectroscopy (EIS) across physiological cortisol concentrations, and cortisol levels were categorized as increasing and decreasing using a weighted K-nearest neighbor (KNN) algorithm. Common issues, such as electrode fouling, low signal-to-noise ratios, and chemical interferences, have been successfully addressed using supervised machine learning models trained on extensive datasets from biosensing technology-based sensors [184].

### 5.2. Optical Sensor-Based Wearables

**Figure 18:**
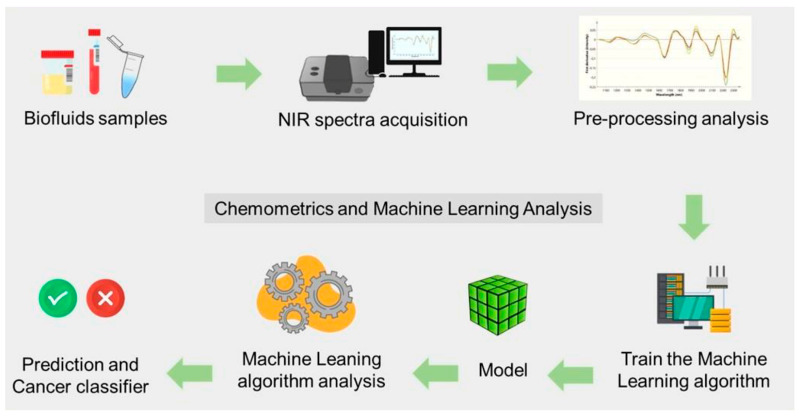
NIR spectroscopy coupled with ML strategy for cancer detection and classification. Reproduced with permission from ref. [187]. Copyright 2023 Elsevier Open Access.

Wearable optical sensor technology, particularly light-based and fluorescence-based systems, has enabled cancer management through continuous, non-invasive monitoring of biomarkers. Conventional methodologies for disease detection usually work when there is a significant concentration of markers in the sample; in contrast, optical sensors are more efficient [185]. Optical spectroscopic methods, such as fluorescence, Raman, MIR, and NIR spectroscopy, may distinguish malignant kidney samples from healthy ones. Bogomolov et al. worked on simplifying the NIR spectroscopy method by substituting an optical sensor that uses a photodiode and light-emitting diodes for conventional high-resolution spectrometry [186]. Optical sensors generally analyze tumor progression or identify early-stage cancer by detecting changes in tissue characteristics using Optical Coherence Tomography (OCT) or Near-Infrared spectroscopy (NIRS). These sensors monitor tissue absorption, reflection, and scattering, providing real-time information on hemoglobin content and tumor oxygenation—two essential parameters for assessing tumor hypoxia. The schematic of NIR spectroscopy and Artificial Intelligence for the early detection of cancer procedure is depicted in Figure 18 [187]. Another similar study found that bioimpedance spectroscopy (BIS) is used to develop a low-cost, simple wearable device for early breast cancer detection. The two sensors operate in tandem to detect variations in the optical and electrical properties of healthy and malignant breast tissue. The NIRS sensor uses multi-wavelength LEDs with optical filters. After processing in a control unit, the aggregated data is sent via Bluetooth to a mobile app.

Organic devices possess unique properties that make them suitable as fabrication materials in domain sensor technology, owing to their durability, lightweight nature, and low power consumption. In their research, Negi et al. compare multilayered organic light-emitting diodes (OLEDs) and triple-hole-block-layer OLEDs to develop an organic LED-based sensor for ovarian cancer detection [188]. Reduced electron-hole recombination in the multilayer OLED enhances light detection, resulting in a six-fold improvement in performance. Most existing techniques are not scalable to incorporate multiple biomarkers. Katchman et al. combined protein microarray technology with an OLED display to produce a compact, high-density, fluorescent, highly sensitive, and multiplexed biosensor, which addresses this issue [189]. Early detection of breast cancer is crucial for higher survival rates, but techniques like MRIs and mammograms are costly and require medical professionals with specialized training. Bhowmick et al. recognized this issue and, to address it, used graphene-based materials to design flexible, long-lasting LEDs for transillumination [190]. This non-invasive technique employs light to detect anomalies in breast tissue. Inspired by human color vision, Ewing et al. investigated a nonspectroscopic infrared optical biomimetic technique for skin cancer diagnosis. In the proposed sensor, three broadband optical filters in the mid-infrared range (2–8 µm) are used to classify tissues as either cancerous or non-cancerous [191]. The double V-groove photonic crystal fiber (PCF)-based sensor is a suitable choice for cancer diagnosis due to its high sensitivity, precise detection capabilities, and ease of manufacture. In contrast to traditional prism-based SPR sensors, this design leverages the unique light-guiding capabilities of PCF sensors, enabling efficient nanoscale light manipulation. In addition to other properties of optical sensors, PCF sensors have a high potential for biosensing and monolithic integration due to their structural flexibility and small size [192].

Fluorescence technology is useful for sensing applications because it can quantitatively correlate the presence of a target analyte with changes in light intensity or wavelength upon excitation by a high-energy photon—the principles of optical fluorescence-based biosensors that utilize small molecules and nanoparticles. Recently, it has been reported how these sensors can be used to find cancer biomarkers in tissues, cells, and bodily fluids. The tissue compartments are based on optical characteristics to differentiate between malignant and healthy skin [167,193]. Using 3D optical coherence tomography (OCT) pictures, an image-processing method extracts the anisotropy factor, scattering and absorption coefficients, and other information to distinguish between healthy skin and skin damaged by BCC. Aptamers, called “chemical antibodies”, are small RNA or DNA molecules with high affinity, stability, and modifiability. Fluorescence sensors based on aptamers provide precise and sensitive identification of cancer biomarkers. Zhao et al. present the latest developments in these sensors, emphasizing their nanostructured architectures and potential uses in early cancer detection, therapy, and prognosis [194]. According to a study by Hussain et al., fluorescence-based sensors employ fluorescent probes to identify cancer-specific compounds or circulating tumor cells (CTCs); these probes release a signal when they attach to the target biomarker [195]. Since nucleic acid probes offer high sensitivity, rapid detection, and adaptability, they have significantly improved cancer management through fluorescence sensing and imaging. These probes can be used to identify abnormal nucleic acids and cancer-associated chemicals, employing sophisticated fluorophores that facilitate early cancer diagnosis and targeted treatment.

Integrating artificial intelligence (AI) with optical sensors has dramatically enhanced their diagnostic accuracy by using data analysis and pattern recognition algorithms. Even in complex biological contexts, machine learning methods can enhance signal processing and simplify the differentiation process of malignant signals from noise [196]. AI and optical sensing provide susceptible, precise, and real-time cancer monitoring systems, which equip wearable sensing platforms to revolutionize cancer surveillance and point-of-care diagnostics [197]. Moreover, this powerful combination can also help to improve the precision of cancer surgeries.

**Figure 19:**
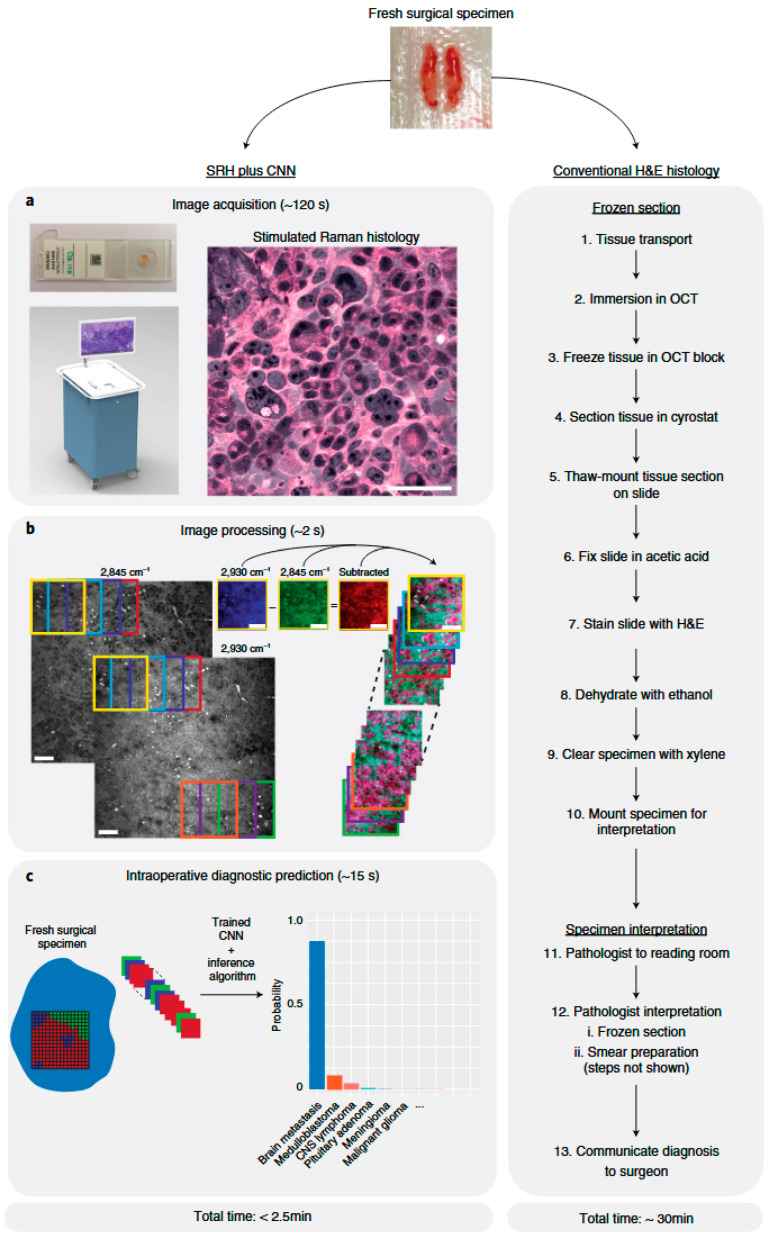
Diagnosis procedure by SRH and DL. Reproduced with permission from ref. [199]. Copyright 2020 Springer Nature Open Access.

A novel method for detecting lung cancer combines integrated optoelectronic biosensors with sophisticated deep learning architectures to reduce noise. Normalization is applied to MRI lung images gathered based on patient medical histories [198]. Key features are distinguished using Lasso regression to effectively identify Fe_3_O_4_ nanoparticles within tumor regions. Bayesian neural networks are used to classify tumors. The stimulated Raman histology (SRH) label-free optical imaging technique is used in conjunction with deep convolutional neural networks (CNNs) to perform automated, near-real-time intraoperative cancer diagnosis [199]. CNNs can help overcome the shortcomings of conventional histologic techniques by employing semantic segmentation to identify tumor-infiltrated areas in SRH images and classify the main histopathologic categories of brain tumors. This method improves the accuracy and timeliness of diagnostics (Figure 19). For cancer theragnostics, optical imaging methods such as optical coherence tomography (OCT), photoacoustic imaging, and fluorescent imaging provide the non-invasive, high-contrast assessment of tumor structures. Xu et al. highlighted in their research how AI improves tumor identification, automated analysis, therapy monitoring, and prognosis prediction through techniques such as deep learning and computer vision, along with specific solutions to address key challenges [196]. Using optical sensors, Nguyen et al. proposed a novel framework that employs the Classification and Regression Tree (CART) algorithm to classify skin cancer into nonmelanoma and melanoma categories [193]. LB orientation angle (α), phase retardance (β), optical rotation (γ), and depolarization indices are some of the optical characteristics obtained from anisotropic biological tissues that were employed as predictors in a Stokes-Mueller matrix formalism. With an accuracy of 92.6%, the CART model proved effective at classifying skin cancers. Similarly, Silver et al. utilized Vibrational optical coherence tomography (VOCT) in their pilot study to distinguish between pigmented and non-pigmented melanomas non-invasively. The results showed that pigmented lesions had greater melanin packing densities [200]. Machine learning approaches have significantly improved sensitivity and specificity by distinguishing skin tumors from healthy skin.

To address issues such as electromagnetic interference and electronic waste, recent advancements have shifted the focus from electrochemical and electrical sensors to optical fiber-based wearable devices. The excellent sensitivity, robustness, and ease of integration with flexible materials of these fiber-based sensors increase their potential for use in cancer-based healthcare applications. Jha et al. reviewed advancements in optical fiber-based wearables, focusing on intensity modulation and wavelength interrogation to identify cancer biomarkers through data analysis using various AI and ML algorithms [98]. Working to construct a fully flexible polymer optical fiber sensor for remote vitals monitoring, Zha et al. designed a stretchable elastomer optical fiber and combined it with polymethyl methacrylate optical fibers [201]. With a maximum strain of over 250%, a high-tensile strain of 100%, and durability of over 500 tests, this intelligent wearable device uses the Beer-Lambert law to enable real-time monitoring of several physiological indicators that aid in cancer identification. The 1-D convolutional neural network is used for data monitoring and analysis. Recently, advancements in nanotechnology have been explored to create smart optical-sensor catheters with enhanced sensing features, including multiplexed fluorescence monitoring and ultrasensitive plasmonic detection [202]. Multimodal imaging and accurate localization are enabled by multifunctional nanocomposites, allowing novel diagnostic insights to be gained through machine learning on large datasets. Lung cancer, a kind of pulmonary cancer, is one of the deadliest types of cancer, and it is treatable only if detected early. Faruqui et al. designed LungNet, an advanced hybrid deep-convolutional neural network that utilizes wearable optical sensor-based medical IoT (MIoT) data and CT scan images to facilitate early diagnosis [203]. By leveraging latent information from both data types, the 22-layer CNN model significantly improves diagnostic precision. After being trained on a balanced dataset of 5.25K pictures, LungNet outperforms other CNN-based classifiers, achieving high accuracy (96.81%) and a low false-positive rate (3.35%). It can accurately divide lung cancer into five groups. Optical sensor integration with various AI and fabrication techniques enhances detection sensitivity and accuracy, making it a powerful tool for early diagnosis and treatment planning, as well as for aiding surgical procedures.

### 5.3. Thermal Imaging Sensors

Thermal imaging, also known as infrared thermography, is a non-invasive cancer-detection method that uses the body’s heat patterns to identify tumor-causing abnormalities. Increased metabolic activities in cancerous tissues frequently result in localized variations in temperature. Wearable thermal sensors can help continuously track these deviations, enhancing early cancer identification and post-therapy follow-up [204]. Moreover, Artificial intelligence (AI), when incorporated into these systems, can further improve them by automating the data analysis, increasing the accuracy of diagnoses, and offering real-time feedback [205]. Infrared (IR) technology is a core component of wearable sensors that utilize thermal imaging. Magalhaes et al. discussed using IR technology to detect infrared radiation from the skin by thermal sensors, which is then correlated with underlying physiological parameters [206]. Typically, wearable thermal sensors use thermopiles or microbolometers to convert infrared radiation into electrical signals, producing thermal maps of the skin’s surface. The data is then examined to find anomalous heat patterns, which are prominent signs of cancerous growth [207].

**Figure 20:**
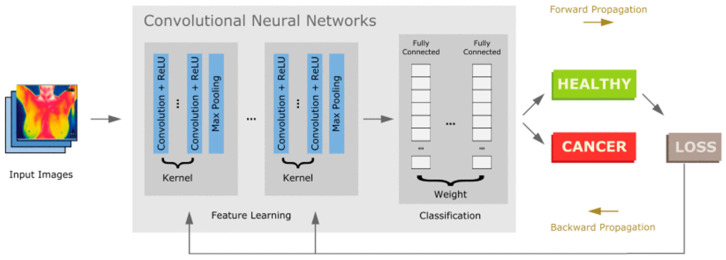
Early identification of Breast Cancer using DL approach CNN and thermograms. Reproduced with permission from ref. [209]. Copyright 2020 IEEE Open Access.

Techniques such as mammography have been used extensively in the past 20 years to diagnose breast cancer. Still, they can cause adverse side effects and false positives, leading to the need for alternative approaches. Infrared digital imaging is a breakthrough approach that suggests precancerous tissues exhibit higher thermal activity than healthy ones. Mambou et al. discussed using this IR approach with Computer-Aided Diagnostic (CAD) systems structured on the hemisphere models to increase diagnostic accuracy [208]. In another similar study, Roslidar et al. analyzed thermal asymmetry in breast tissues as a critical symptom of malignancy. According to reports, thermal imaging sensors can perform exceptionally well at capturing such symmetric differences. Moreover, adopting these sensors provides a non-invasive, physical contact-free medical imaging screening technique that does not harm breast tissues (Figure 20) [209].

In their study, Aggarwal et al. utilized the Gray Level Co-occurrence Matrix (GLCM) to extract texture data with descriptors such as energy, correlation, contrast, and color information from RGB, HSV, and LAB spaces [210]. Several machine learning techniques, including k-Nearest Neighbors (KNN), decision trees, and linear discriminant analysis, were employed to classify breast cancer in thermal images. The KNN classifier’s superior performance suggests it could aid in building a computer-aided preliminary screening system for cancer images. The concept of physics-informed neural networks is explored to model the thermal behavior associated with breast cancer. The temperature field or thermal conductivity is determined using the Adam optimizer in TensorFlow, which minimizes losses computed at random locations based on boundary conditions and the governing bioheat equations. Backpropagation is used to calculate derivatives in the bioheat equation [211].

Khan et al. designed wearable thermal sensors to measure breast temperature and generate breast thermograms continuously [212]. The researchers employed machine learning to develop a customized classifier based on the ResNet architecture, incorporating key components from GoogleNet, including 2D CNNs and activation functions. To improve the classification accuracy of the designed system, the hybrid model included max pooling and batch normalization after each convolution. Using infrared thermography, Fernandez-Ovies et al. performed an initial evaluation of convolutional neural networks (CNNs) for early breast cancer diagnosis. They examined many CNN designs using the FastAI and PyTorch packages, where ResNet34 and ResNet50 achieved 100% predicted accuracy. These deep learning models performed well in early cancer detection using easily applicable computational technologies [205]. A Personalized risk assessment framework, i.e., Thermalytix Risk Score (TRS), is designed for widespread early breast cancer identification [213]. TRS analyses thermal images using AI to produce a breast health risk score, computed from two sub-scores: vascular and hotspot. The vascular score evaluates asymmetrical vascular activity, while the hotspot score reflects irregular heat patterns on the skin. By analyzing medically interpretable characteristics, machine learning algorithms can assess breast tissue metabolic activity and even identify potential cancers in asymptomatic women.

### 5.4. Ultrasound-Based Wearable Sensors

**Figure 21:**
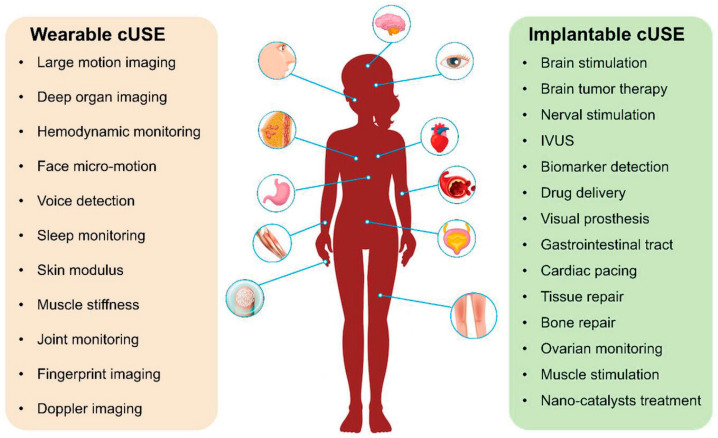
Wearable and implantable conformable ultrasound electronics (cUSE) for various healthcare uses. Reproduced with permission from ref. [214]. Copyright 2023 Wiley-VC Open Access.

The non-invasive nature of ultrasound-based technologies, their capability for real-time imaging, and the use of non-ionizing radiation, and their potential to identify structural abnormalities in tissues have made them widely used in cancer diagnostics [214]. On the other hand, the bulky size of conventional ultrasound equipment makes it difficult to provide personalized cancer care and continuous monitoring. However, the emergence of wearable technologies is stretching the limits of traditional ultrasound, with an urgent requirement to create real-time, continuous monitoring systems that incorporate ultrasound into wearable cancer care equipment. Several wearable and implantable conformable ultrasound electronics (cUSE) have been developed for various healthcare management, including cancer (Figure 21). Huang et al. have highlighted developments in soft electronics and miniaturization, and have provided an overview of materials, fabrication processes, beamforming, and applications for wearable ultrasonic devices. The researchers have categorized wearable ultrasonic devices into three types: rigid, flexible, and stretchable, each with unique fabrication and design techniques [215].

**Figure 22:**
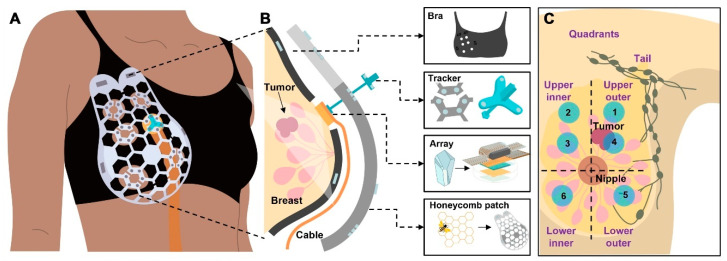
(**A**) Schematic and (**B**) exploded view of cUSBr-Patch on the body with main components (bra, tracker, array, and honeycomb patch), and (**C**) Schematic of breast quadrants and the positions of circular regions that align with the patch openings and circular holes in the bra. Reproduced with permission from ref. [216]. Copyright 2023 American Association for the Advancement of Science Open Access.

In a groundbreaking study, Du et al. created a comfortable, wearable ultrasound breast patch (cUSBr-Patch) that enables reliable, operator-independent imaging of the whole breast [216]. The thin-film ultrasound transducers in the patch were embedded in a soft substrate that adhered to the skin, enabling cancer screening and real-time imaging without a handheld device. Moreover, the honeycomb-shaped architecture enables deep, multi-angle, and extended breast scans when paired with a phased-array of piezoelectric crystals and a tracker (Figure 22). Similarly, Zou et al. designed a wearable and portable anticancer device that uses a specially designed multiplexed ultrasonic patch array (CWUS Patch) for continuous sonodynamic therapy [217]. This technique utilizes ultrasound to precisely target the lesion site and trigger sonosensitizers, which produce toxic reactive oxygen species (ROS). Its deep penetration causes tumor cell apoptosis, and its real-time control improves flexibility and precision. Chen et al. have introduced an elastic and flexible ultrasonic transducer (FSUT) that can adapt to curved human skin while still producing high-quality images [218]. This invention utilizes screen-printed silver nanowires (AgNWs) to achieve long-lasting mechanical and electrical connections over a composite elastic substrate. FSUTs are ideal for wearable devices due to their exceptional tensile strain (≥110%), flexibility (R ≥ 1.4 mm), and lightweight design (≤1.58 g). Additionally, the accuracy of the prediction power of these devices is being further improved by the application of artificial intelligence (AI) approaches, which makes them useful in contemporary healthcare [219,220]. The model analyzes ultrasound images by automatically identifying suspicious spots and differentiating between normal and malignant tissues. It further distinguishes between malignant phases and benign disorders, such as fibroids, facilitating early identification and improving treatment outcomes. Various AI-based wearable sensing devices for Cancer management and their features, such as the type of wearable device, type of AI, target analyte, limit of detection, detection range, selectivity, specificity, pros and cons, are summarized in Table 3.

In recent years, the scope of AI-based ultrasound imaging for diagnosing focal liver lesions and diffuse liver illnesses was explored, determining the severity of non-alcoholic fatty liver, staging liver fibrosis, and identifying benign and malignant liver tumors [221]. Moreover, researchers also discussed the process of identifying pancreatic cancer, distinguishing between gastric mesenchymal tumors, and forecasting preoperative tumor deposits in rectal cancer. Artificial intelligence has also been utilized in endoscopic ultrasonography for the diagnosis and treatment of gastrointestinal disorders. These developments enhance therapy outcomes and facilitate early diagnosis. Tagnamas et al. introduce a sophisticated multi-task framework that uses a hybrid CNN-ViT architecture with an MLP-Mixer to segment and classify tumors in breast ultrasound (BUS) pictures [222]. The technique employs a dual-encoder architecture: an updated ViT encoder utilizes self-attention to extract high-level features, while an EfficientNetV2 backbone captures local image information. To improve the integration of these features and produce accurate segmentation maps, a Channel Attention Fusion (CAF) module is presented. The segmented tumors are then categorized as benign or malignant using an effective MLP-Mixer classifier, a new method for classifying lesions in BUS images.

**Table 3:** Summary of literature on AI-based wearable sensing technologies for cancer management.

Type of Wearable Sensor	AI/Algorithm Used	Target Analyte	Limit of Detection	Detection Range	Selectivity	Specificity	Pros	Cons	Ref.
Sweat biomarker monitoring patch	Machine learning-based pattern recognition for multi-analyte data integration (described conceptually in review)	Sweat metabolites and hormones	Varies by analyte: Lactate ~0.35 µM; Cortisol ~0.1 pg/mL; Glucose ~35 µM	1 µM–15 mM (depending on analyte)	High with enzyme/MIP-based sensors	Depends on immobilization method (LOx, GOx, Ab-based)	Non-invasive, real-time, multi-analyte detection, compatible with AI data analysis, wireless capability	Biofouling, enzyme degradation, sensor drift, and limited sweat volume in low perspiration conditions	[177]
Osmotic hydrogel–microfluidic electrochemical lactate patch	Data-driven modeling for activity recognition and lactate trend prediction (custom AI model used for calibration drift correction)	Lactate	350 nM	Up to 15 mM	High (enzyme-based)	Specific to lactate oxidase substrate	Zero-power operation; continuous monitoring at rest and exercise; ultra-low power wireless module (0.706 mW)	Enzyme degradation over time; humidity-dependent response; limited long-term stability	[178]
OLED-based organic photonic biosensor	AI-assisted signal deconvolution and light intensity correlation model (image pattern recognition)	Ovarian cancer-related bioluminescent markers	13–29 mA photocurrent at 420–440 nm (relative intensity-based detection)	Optical wavelength 400–500 nm	High spectral specificity	Light wavelength-based signal discrimination ensures selectivity	Flexible, low-cost, biocompatible, lightweight; suitable for optical AI processing	Needs calibration; sensitive to ambient light; lacks multiplexing capability	[188]
Flat-panel OLED display-based multiplexed immunosensor	Neural network-based fluorescence signal recognition for multiplexed biomarker classification	HPV-related IgG antibodies	10 pg/mL (for IgG antibodies)	10 pg/mL–10 µg/mL	High with antibody–antigen binding	Multiplexed detection reduces false positives via AI pattern discrimination	Compact, disposable, low-cost, high-throughput multiplexing; scalable via display manufacturing	Complex calibration; needs fluorescence reference standardization	[189]
AI-enhanced optical polarization sensor for skin cancer detection	Classification and Regression Tree (CART) algorithm	Skin cancer lesions (melanoma, SCC, BCC)	Not applicable (classification-based system)	Optical feature extraction model-based	High (92.6% accuracy in distinguishing cancer types)	Accurate classification of malignant vs. non-malignant samples	Non-invasive, interpretable AI model, real-time diagnosis	Limited sample diversity; optical noise under skin curvature	[193]
Vibrational Optical Coherence Tomography (VOCT) Patch	Machine Learning Classification (unspecified model)	Pigmented vs. non-pigmented melanoma lesions	N/A (classification accuracy based)	80–250 Hz frequency response range	High (78–90% accuracy for lesion differentiation)	Up to 90%	Non-invasive, quantitative, real-time detection of melanoma phenotypes	Requires calibration; limited large-scale clinical validation	[199]
Infrared Thermal Imaging System (contactless wearable-assisted imaging)	Deep Learning Image Classification Models (DNN, CNN, SVM)	Breast cancer lesions	Temperature resolution ~0.05 °C	Skin surface temperature 30–40 °C	Distinguishes malignant vs. benign tumors based on thermal variance	Up to 94% with a CNN-based classifier	Non-contact, radiation-free, suitable for early cancer screening	Affected by ambient temperature; requires calibration and a trained dataset	[208]
Medical Infrared Thermography (IRT) for skin neoplasms	Pattern recognition and machine learning-based classification	Skin cancer (melanoma, BCC, SCC)	Thermal sensitivity < 0.1 °C	30–42 °C	High when combined with ML	Improved diagnostic accuracy via dynamic thermal analysis	Contactless, safe, non-ionizing, real-time detection	Sensitive to environment; limited depth resolution	[206]
Conformable Ultrasound Breast Patch (cUSBr-Patch)	AI-based image reconstruction and lesion classification (CNN-enhanced)	Breast tissue cysts and lesions	~0.3 cm cyst	Up to 30 mm tissue depth	High acoustic contrast (3 dB)	Clinical-level accuracy with AI-assisted analysis	Non-invasive, comfortable, real-time deep tissue monitoring	Complex fabrication; limited penetration beyond 30 mm	[218]
Fully Integrated Conformal Wearable Ultrasound Patch (CWUS)	AI-based control and optimization for ultrasound power modulation	Cancerous tissue (tumor apoptosis induction)	N/A (therapy-based system)	Deep tissue penetration (>30 mm)	High, focused ultrasound localization	Precise tumor targeting via AI-controlled focusing	Continuous, non-invasive tumor treatment; adaptive control via AI	Requires power management and safety calibration	[218]
Hybrid Deep-CNN LungNet System (IoT-integrated)	Hybrid Deep-CNN (LungNet)	Lung cancer detection and stage classification	N/A (classification accuracy-based)	Stage 1A–2B classification	96.81% classification accuracy	Low false positive rate (3.35%)	The hybrid IoT-CNN model integrates real-time physiological data with imaging	Centralized server required; data privacy considerations	[203]
CNN-Transformer Breast Ultrasound Classifier	Hybrid CNN + Vision Transformer (ViT) + MLP-Mixer	Breast tumor segmentation and classification	N/A (accuracy-based segmentation)	Tumor size variability from ultrasound imagery	High (Dice 83.42%)	86% classification accuracy	High interpretability; captures long-range dependencies; robust tumor segmentation	High computational load; training dataset requirement	[223]

Table 4 summarizes the AI-based algorithms most effective for each disease application, with high reproducibility. Support vector machines (SVMs) have demonstrated excellent performance in categorizing wearable respiratory signals for COVID-19. At the same time, convolutional neural networks (CNNs) and recurrent neural networks (RNNs) have proven most successful for cough sound analysis and chest imaging [223]. For diabetes, Deep learning architectures, especially CNNs in conjunction with long short-term memory (LSTM) networks, have been extensively used to analyze continuous glucose monitoring (CGM) data. However, Random Forest (RF) and gradient boosting models have proven to be effective in managing multivariate physiological and electrochemical signals [224]. SVM and k-nearest neighbor (KNN) are still helpful for small-scale wearable biosensor datasets, whereas CNNs and hybrid CNN–transformer models dominate imaging-based detection (ultrasound, thermal, and wearable patches) for cancer management [225].

Benchmark datasets that cover both optical sensor modalities (such as dermoscopy, OCT, and hyperspectral imaging) and biosensing data (such as proteins, enzymes, and metabolites from sweat, saliva, or interstitial fluid) that can support the modeling and learning of AI-based wearable biosensors for cancer detection are included in the revised manuscript. These datasets serve as the basis for training and validating AI algorithms. For a list of such representative resources (Table 5).

**Table 5:** The datasets serve as the basis for training and validating AI algorithms.

Dataset	Biological Fluid or Modality	Cancer Biomarkers or Features	Data Type	Refs.
Salivary Biomarker Datasets	Saliva	IL-6, IL-8, TNF-α, MMP-9, CA-125	Proteomic and cytokine patterns for breast and oral cancer	[226,227]
SalivaDB dataset	Saliva	miR-146a	proteins, metabolites, microbes, micro-ribonucleic acid (miRNA), and human genes	[228]
Dataset for Oral Cancer Cytology (OSCC)	oral swabs or images of cells	Oral squamous cell carcinoma-related cellular abnormalities	Images of cytology with annotations	[229]
Cancer Pain Multimodal Dataset	Physiological indicator	Physiological indicators of pain in cancer	Time-series behavioral and wearable signals	[230]
HAM10000 Dataset	Optical dermoscopy images	Skin lesions with pigmentation, such as melanoma	High-resolution dermoscopy images	[231]
International Skin Imaging Collaboration (ISIC) Archive	Dermoscopy Images	Skin cancer (BCC, SCC, melanoma)	An extensive public collection of dermoscopy images	[232]

However, like dietetic (OhioT1DM dataset for glucose prediction) and COVID (open datasets on physiological signals such as heart rate, SpO_2_, and respiratory pattern), publicly available benchmark datasets for wearable cancer sensors remain very limited [233]. Cancer-related medical imaging (TCGA, TCIA) and multi-omics repositories are the datasets helpful for AI model training for cancer detection [234,235]. However, they are not suitable for wearable sensing. Only a few studies have reported small-scale datasets from wearable sensing devices, including physiological signals, thermal profiles, activity monitoring, and biomarker surrogates in cancer patients. But they are not standard benchmark datasets. Therefore, it is essential to create standardized, large-scale, and open benchmark datasets for wearable cancer-sensing devices. It would be helpful for rapid AI development, for comparing algorithms, and for fostering interdisciplinary collaboration among clinicians, data scientists, and engineers.

## 6. Limitations and Challenges

Moreover, the ethical implications, such as privacy and confidentiality, informed consent, algorithmic bias and fairness, data ownership and control, and equitable access to wearable data collection, are especially sensitive in health conditions like cancer, and need to be considered. There is a risk of unauthorized access and potential misuse of patient data and treatment information. There is a need for transparent data sharing policies, where patients are fully aware of how their data will be collected, analyzed, and stored [236]. Further biased datasets also need to be addressed to prevent unequal healthcare outcomes across diverse patient populations. The patient should have ownership of their data to prevent its misuse, and their consent must be obtained before it is used. Further advanced AI-based wearable technologies should not create a gap between different economic classes.

Moreover, there are gaps in the development of AI-based wearable sensing technology, including scalability, data security, and clinical validation. Scalability poses significant challenges due to the high cost of hardware, limited access in low-resource settings, and the need to integrate with existing healthcare infrastructures. These limitations need to be overcome through the development of cost-effective AI-based wearable devices that integrate seamlessly into the existing healthcare system [237]. Furthermore, it is crucial to validate AI-powered wearable devices clinically through extensive clinical studies. However, several safety considerations need to be addressed before clinical validation to avoid adverse effects in patients. The reliability, reproducibility, and regulatory approval pathways of AI-based wearable devices need to be thoroughly investigated before their adoption in the healthcare system [238].

Future research should focus on developing AI-based algorithms and models capable of analyzing and predicting trends in various pathways involved in cancer. Interdisciplinary collaboration is one way to overcome the multiple challenges involved in developing AI-based wearable sensing technologies. Hence, the experts in the field of AI, medical devices, design, clinicians, engineers, data scientists, regulatory authorities, and policy makers need to work together in collaboration to bridge the gap between technology development, validation, clinical translation, and ethical consideration [239].

## 7. Conclusions and Future Perspectives

AI-based wearables sensing technology is a promising frontier in the management of diseases such as COVID-19, Diabetes, and Cancer. These technologies provide benefits such as real-time, continuous, and non-invasive tracking, enhancing early-stage disease detection and enabling effective treatment, thereby limiting further complications. The integration of advanced AI algorithms with wearable sensing techniques enables precise, accurate, sensitive, and efficient data analysis, enabling timely interventions and significantly reducing healthcare costs, clinic visits, the demand for healthcare professionals, and the need for manpower and caretakers. AI-based wearable sensing devices primarily use electrochemical, colorimetric, optical, and pressure/strain sensing techniques. In COVID-19 management, various AI-based wearables have been developed to monitor respiratory rate, temperature, heart rate, heart rate variability, and oxygen saturation. These are the key parameters for determining COVID-19 status. Wearable sensors for COVID-19 management played a crucial role in managing patients with limited access to medical professionals during the pandemic. For diabetes management, various technologies have been developed, including continuous glucose monitoring sensors, AI-driven insulin pumps, closed-loop systems, sweat-based sensors, and AI-integrated applications like smartwatches and fitness trackers. These technologies are significant indicators of diabetes status. Glucose-sensing wearable devices and smartphone applications play a crucial role in managing patients with diabetes. For Cancer management, various AI-based wearable devices have been developed to monitor cancer biomarkers. AI-based thermal imaging wearable sensing devices have been designed to capture images of cancer sites and differentiate between healthy and cancer tissue sites based on thermal imaging processing and analysis-driven AI. Recently, ultrasound-based wearable sensing technology has made significant progress due to its non-invasive, adaptable, patient-friendly, and deep-tissue-penetrating efficacy. The groundbreaking advancement in AI-based cancer management involves the development of a comfortable wearable ultrasound breast patch, comprising a bra, a tracker, an array, and a honeycomb patch. It is capable of independent imaging of the whole breast and monitoring the breast cancer status. This technology has great potential for application to other cancers.

Among recent developments, a significant number of AI-based wearable sensing devices have been developed for the management of diabetes and COVID-19, compared to Cancer. This is mainly due to various challenges associated with cancer compared to COVID-19 and diabetes. Pathophysiology, symptoms, biomarkers, disease condition, disease progression, disease stage, potential diagnostic mechanisms, and therapeutic strategies are well understood in the cases of COVID-19 and diabetes. Hence, several AI-based wearable sensing devices have been developed and are available in the market for the management of COVID-19 and diabetes. However, the pathophysiology of cancer is very complex; there are several pathways involved in cancer progression, the progression in cancer is swift, and it is challenging to manage cancer just by monitoring one or two biomarkers responsible for cancer. Hence, for cancer management, AI-based wearable sensing devices have the potential to collect, analyze, adjust, and visualize data, providing clear, accurate information on the molecular pathways and mechanisms involved in cancer progression. The overall information helps analyze the status of cancer and the expression levels of multiple biomarkers that are overexpressed or suppressed by cancer. Hence, the currently developed AI models need to be significantly improved to monitor biomarkers, recognize various pathways, and provide an exact status of Cancer.

## Figures and Tables

**Table 4:** Summary of the various AI algorithms that are most effective for specific diseases with reproducibility.

Disease	Target Parameter for Wearable Sensor	Significant AI Algorithm
COVID-19	Imaging, Respiration, and Cough	CNN, RNN, SVM
Diabetes	Electrochemical Patches and CGM	CNN, LSTM, RF, Gradient Boost
Cancer	Ultrasound, Thermal, Sweat Patches	CNN, CNN–CNN-Transformer, SVM, KNN

## Data Availability

No new data were created or analyzed in this study.
